# Supplementary material for: Nanoparticles Based on Novel Carbohydrate-Functionalized Polymers
Source: Molecules. 2020 Apr 10;25(7):1744. doi: 10.3390/molecules25071744 (PMC7180923; doi:10.3390/molecules25071744)
Supplement: Supplementary file 1 [file molecules-25-01744-s001.pdf]

Nanoparticles Based on Novel Carbohydrate-Functionalized Polymers

Cláudia D. Raposo[1,†], Cristiano A. Conceição[1,†], M. Teresa Barros[1,*]

[1] LAQV, Requimte, Chemistry Department, NOVA School of Science and Technology, Universidade Nova de Lisboa, 2829-516, Caparica, Portugal
* Correspondence: mtb@fct.unl.pt; Tel.: 0035 212 948 300
† These authors contributed equally to this work, as first authors.

Compounds characterization: ^{1}H, ^{13}C NMR, ESI-MS and MALDI-TOF spectra

*Ethyl 3-(7-hydroxy-4,8-dimethyl-2-oxo-2*H*-chromen-3-yl)propanoate (**1**)*

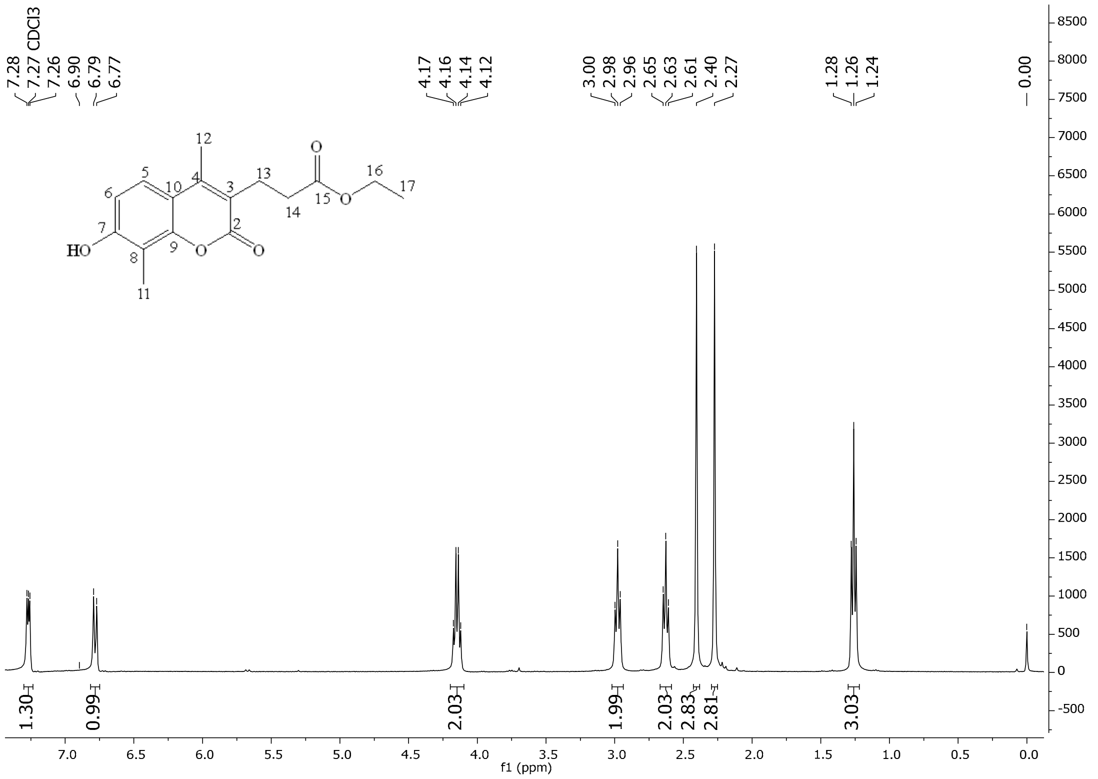

S01- ^{1}H NMR (400 MHz, $CDCl_3$) spectrum of compound **1**.

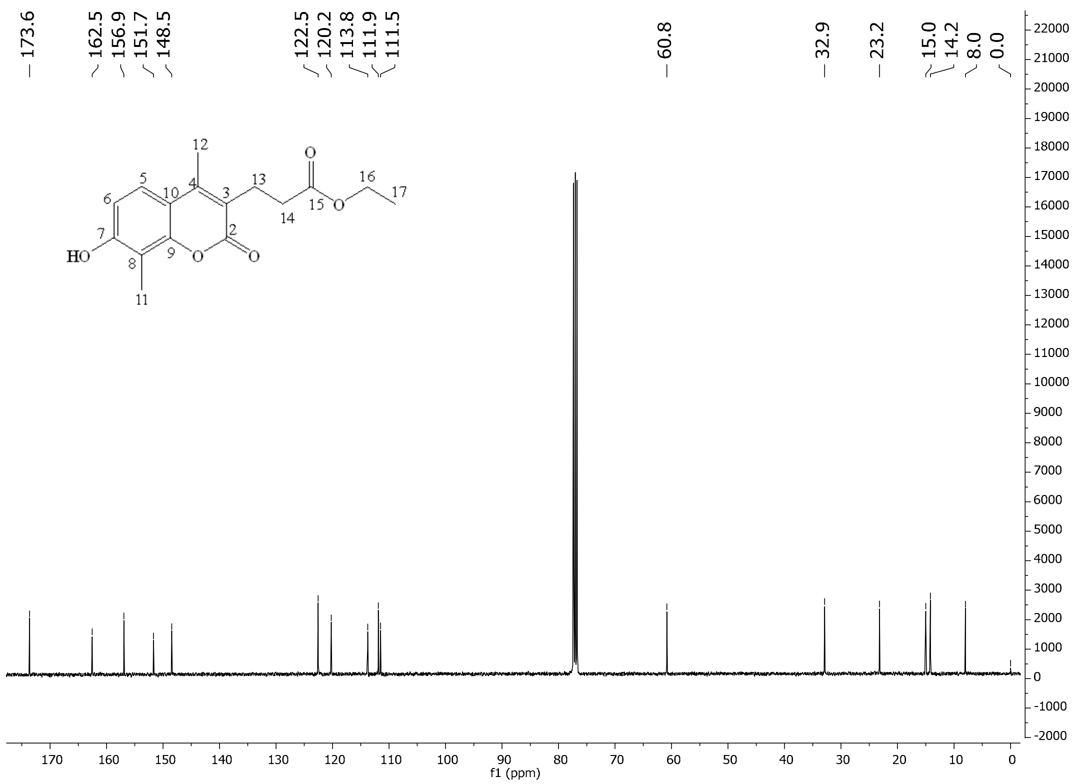

S02- ^{13}C NMR (101 MHz, $CDCl_3$) spectrum of compound **1**.

*Ethyl 3-(4,8-dimethyl-2-oxo-7-(prop-2-yn-1-yloxy)-2*H*-chromen-3-yl)propanoate* (**2**)

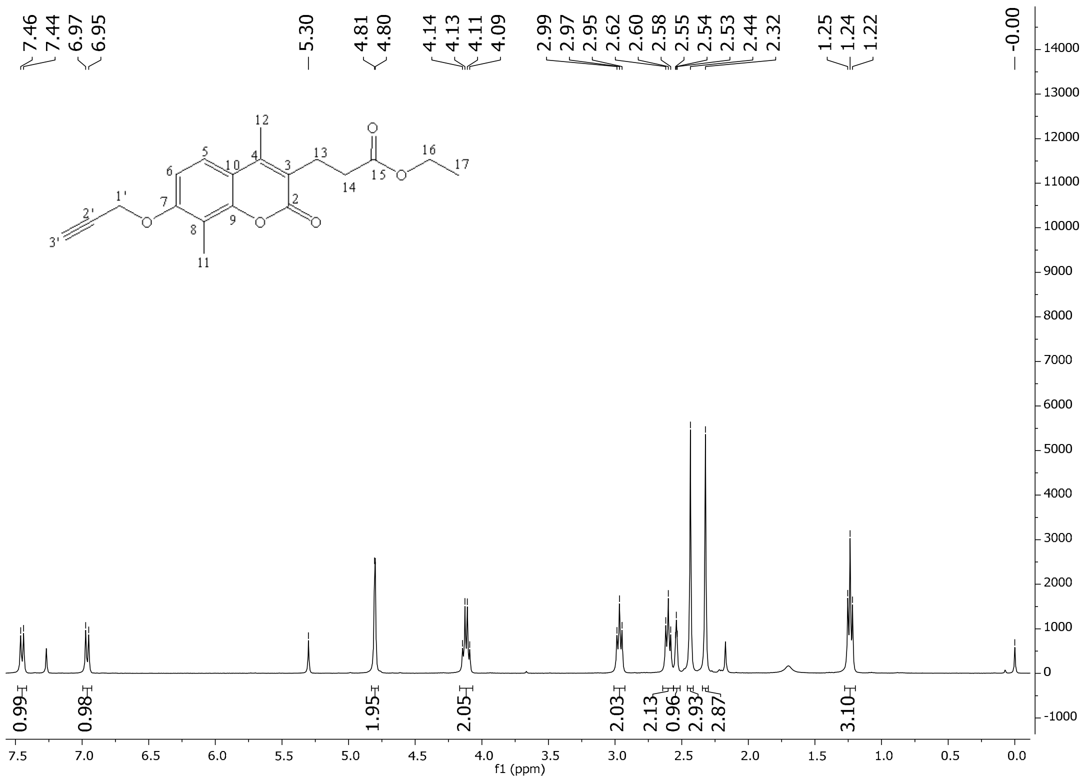

S03- ^{1}H NMR (400 MHz, $CDCl_3$) spectrum of compound **2**.

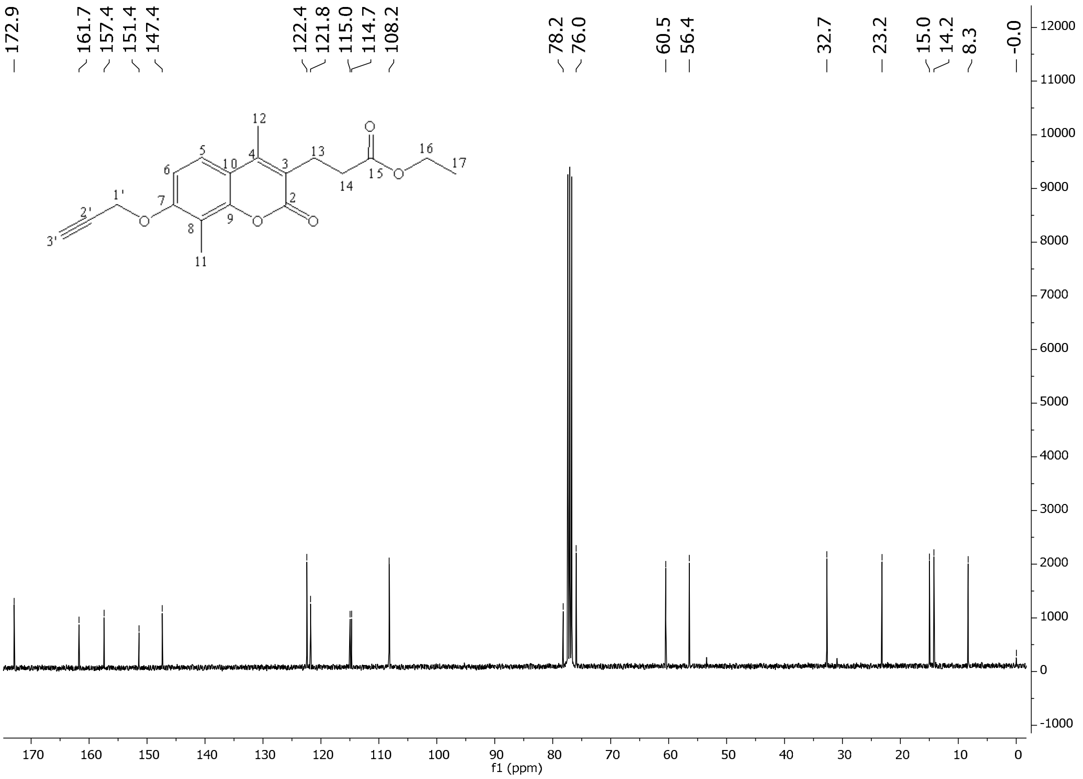

S04- ^{13}C NMR (101 MHz, $CDCl_3$) spectrum of compound **2**.

*3-(4,8-Dimethyl-2-oxo-7-(prop-2-yn-1-yloxy)-2*H*-chromen-3-yl)propanoic acid (***3***)*

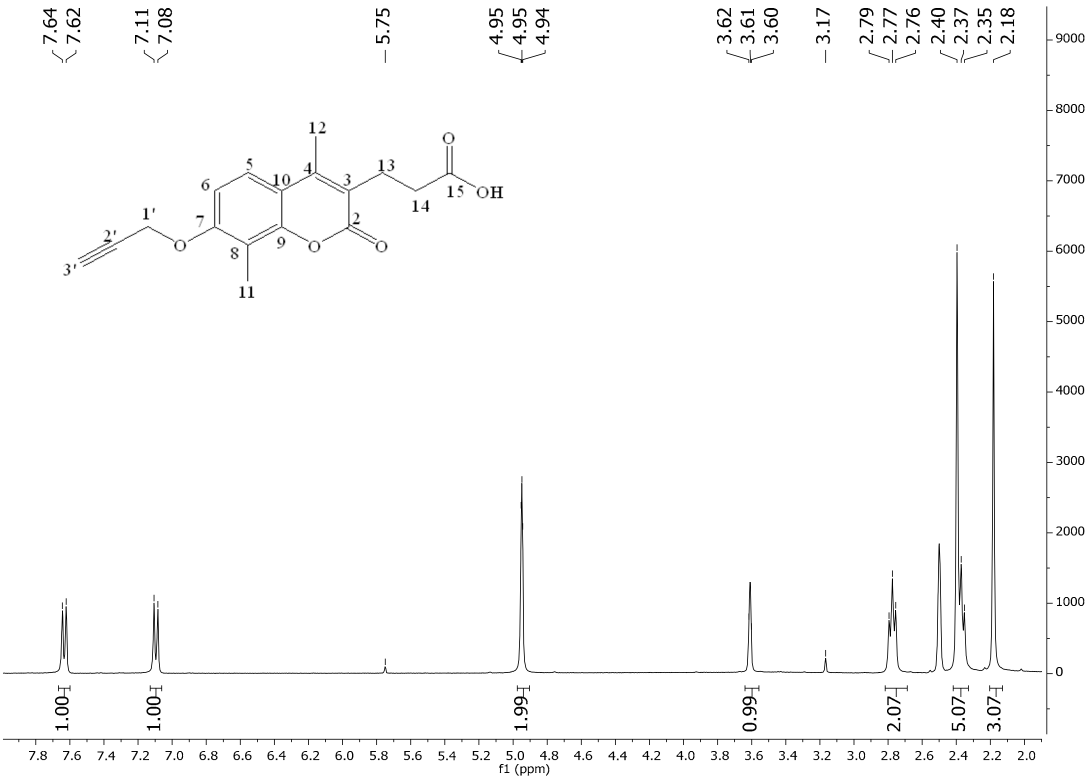

S05- ^{1}H NMR (400 MHz, DMSO-d_6) spectrum of compound **3**.

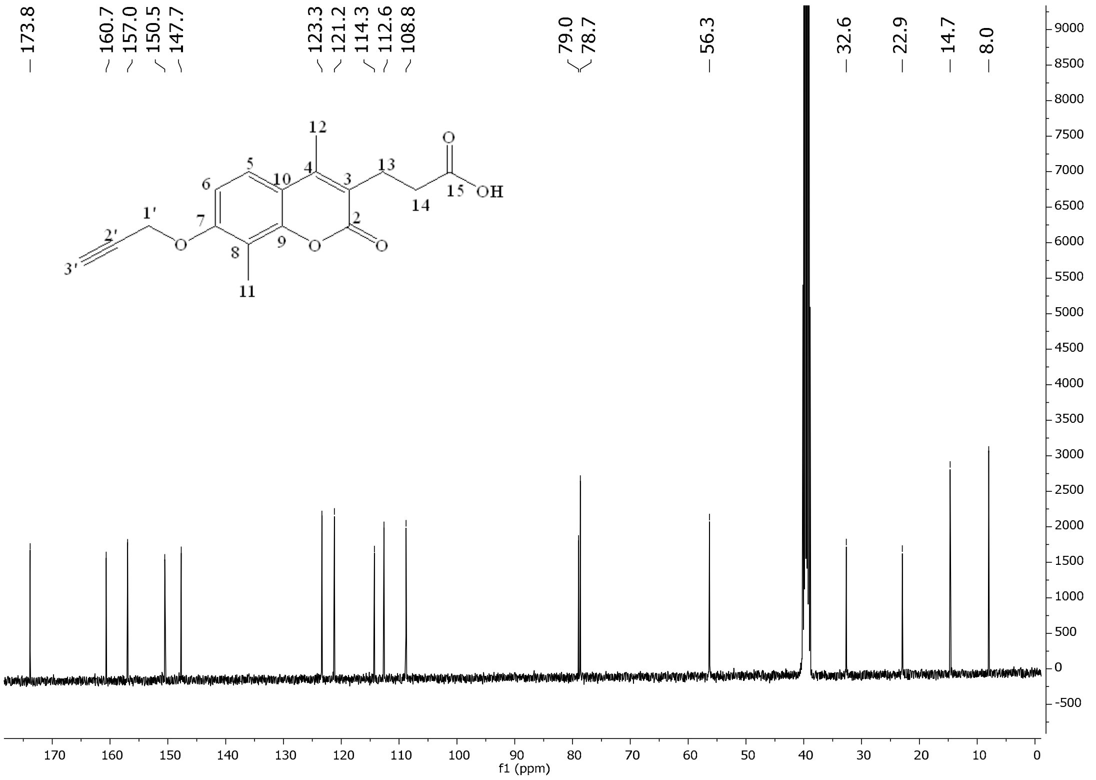

S06- ^{13}C NMR (101 MHz, DMSO-d_6) spectrum of compound **3**.

*4-methyl-7-(prop-2-yn-1-yloxy)-2*H*-chromen-2-one* (***6***)

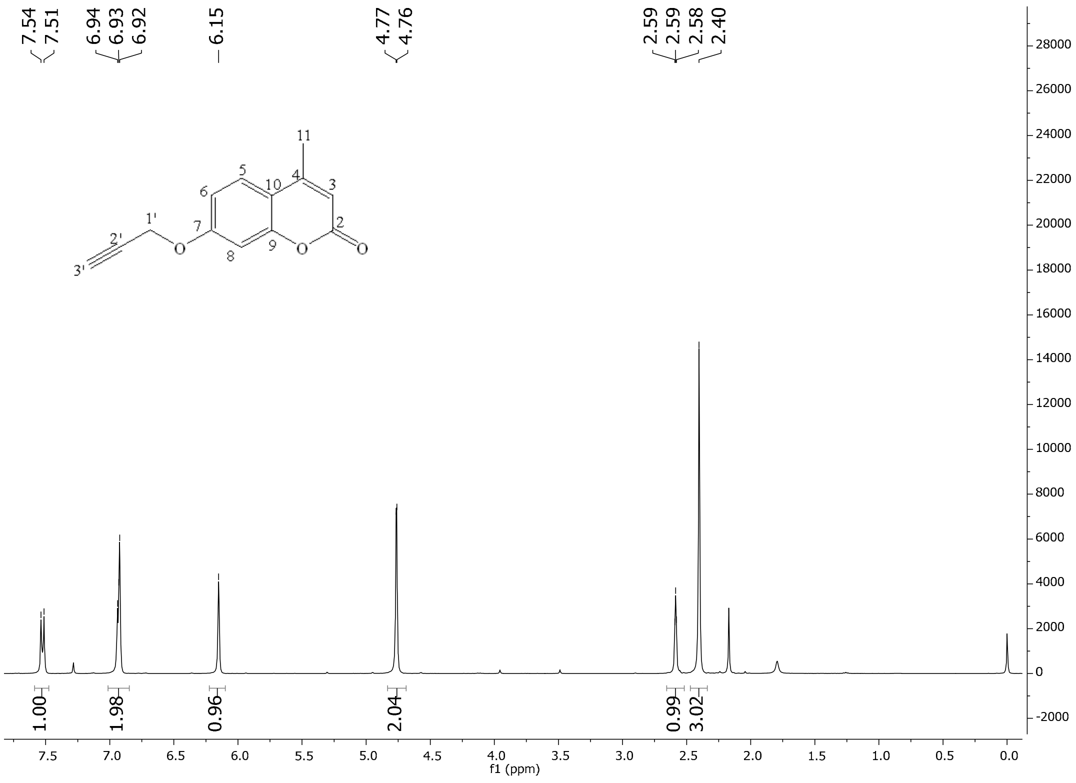

S07- ^{1}H NMR (400 MHz, $CDCl_3$) spectrum of compound **6**.

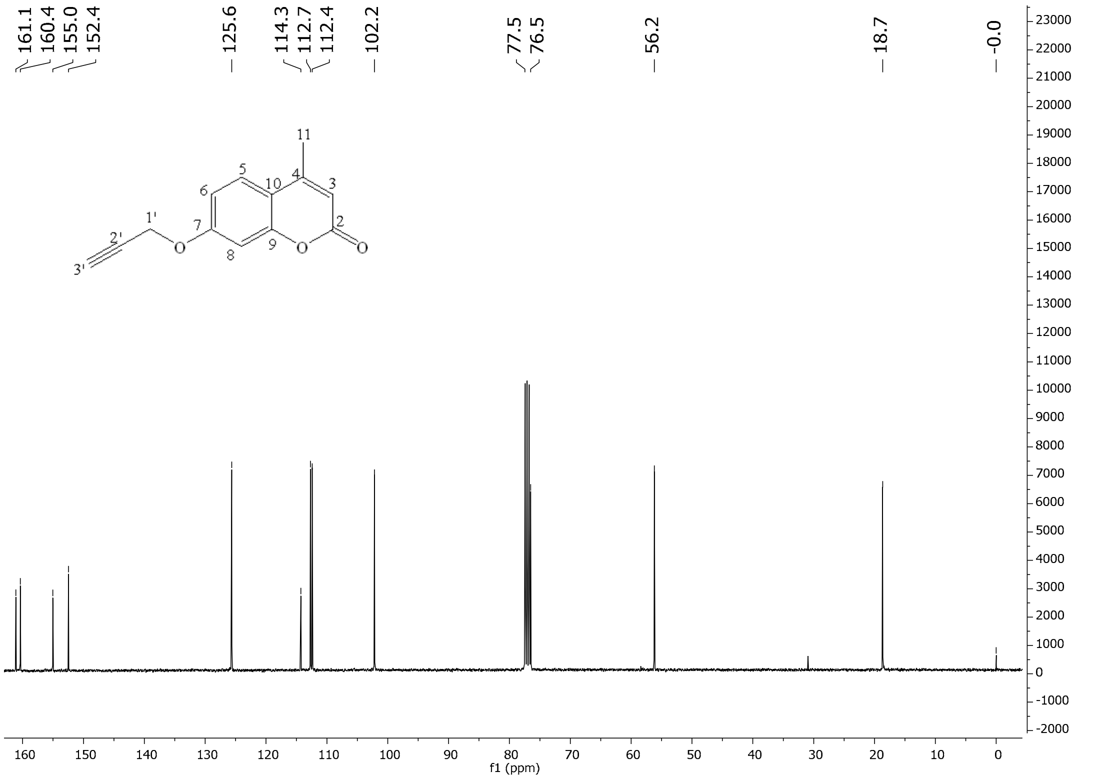

S08- ^{13}C NMR (101 MHz, $CDCl_3$) spectrum of compound **6**.

*2-Azidoethanamine (***7***)*

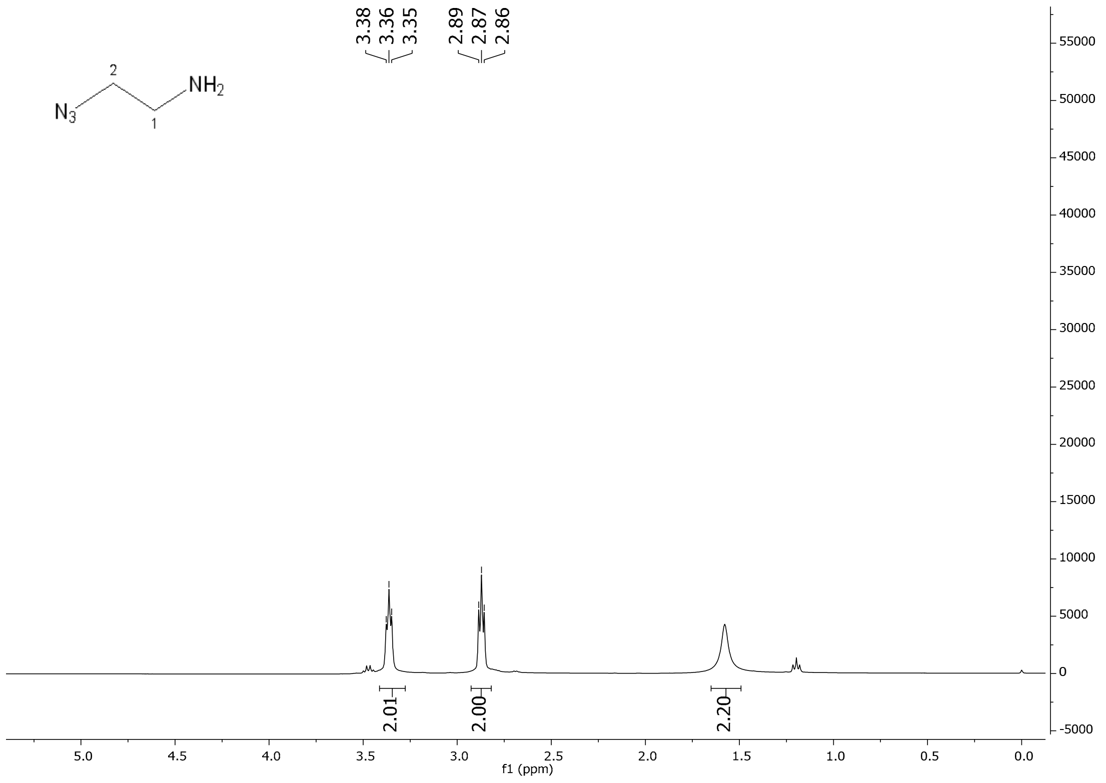

S09- ^{1}H NMR (400 MHz, $CDCl_3$) spectrum of compound **7**.

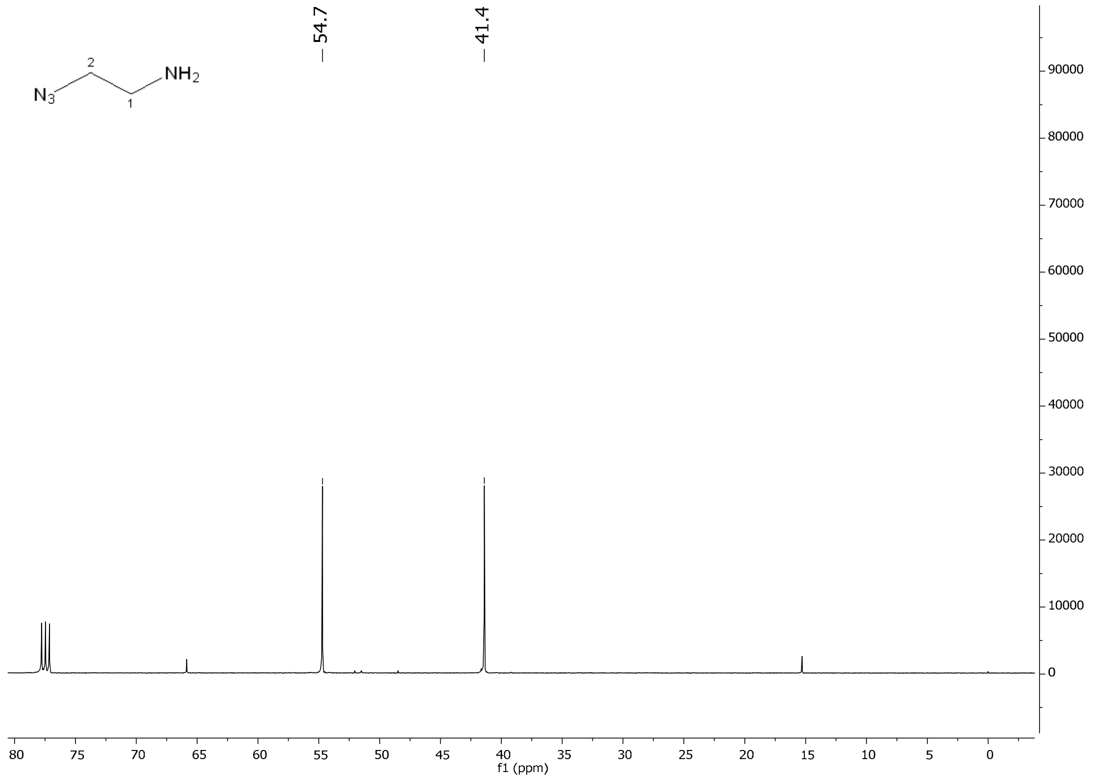

S10- ^{13}C NMR (101 MHz, $CDCl_3$) spectrum of compound **7**.

*7-((1-(2-Aminoethyl)-1*H*-1,2,3-triazol-4-yl)methoxy)-4-methyl-2*H*-chromen-2-one (**8**)*

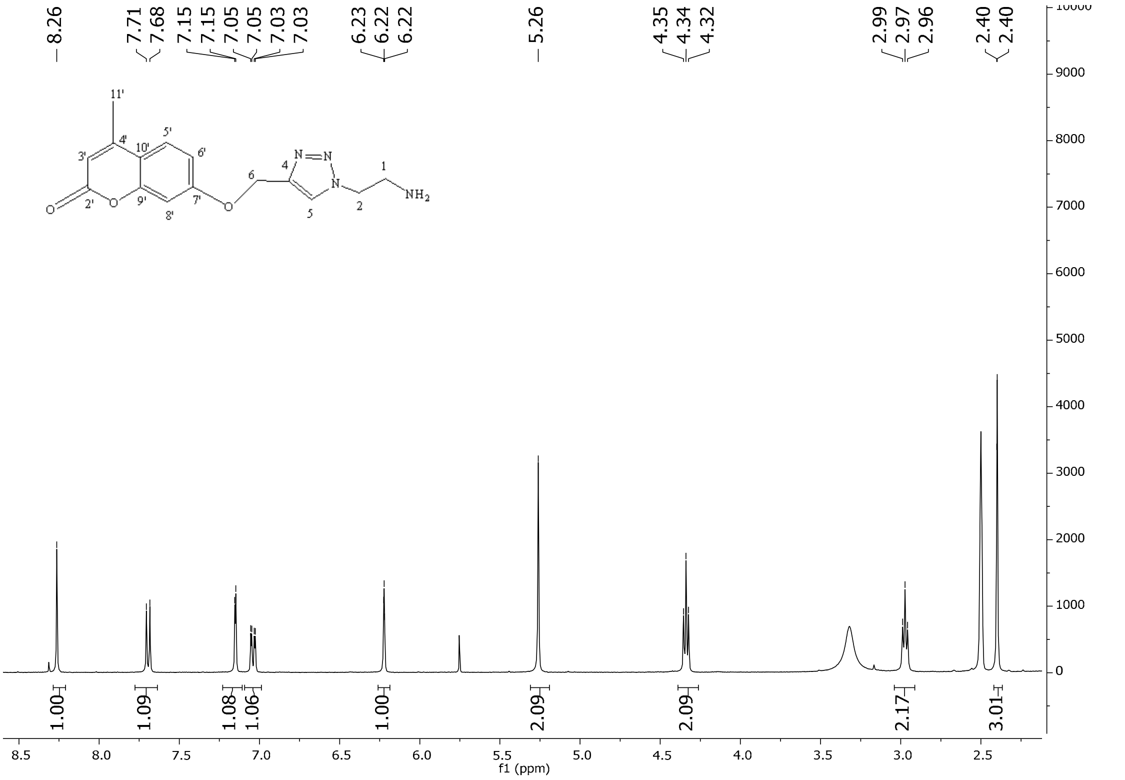

S11- ^{1}H NMR (400 MHz, DMSO-d_6) spectrum of compound **8**.

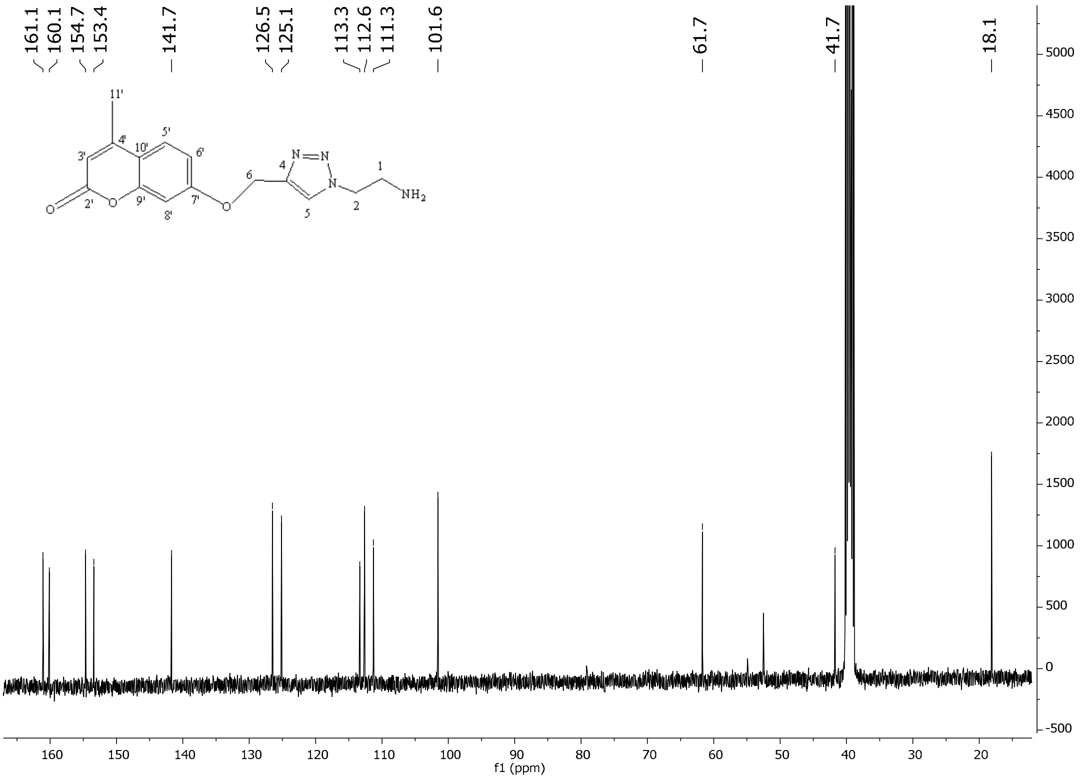

S12- ^{13}C NMR (101 MHz, DMSO-d_6) spectrum of compound **8**.

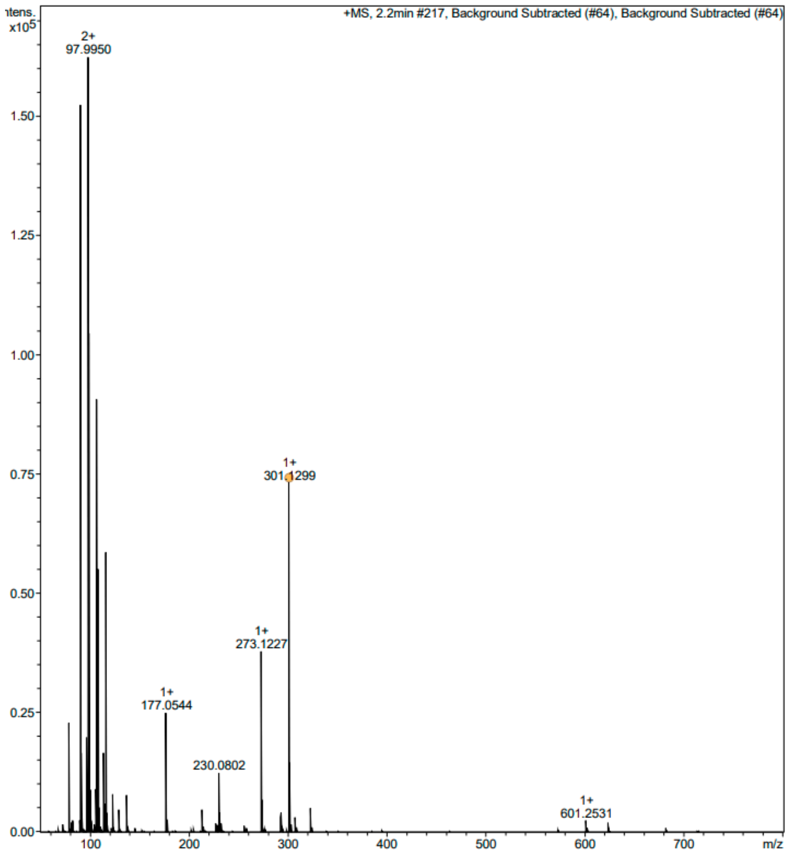

S13- ESI-MS spectrum of compound **8**.

*1-O-Propargyl-2,3,4,6-tetra-O-acetyl-D-glucopyranoside (**12**)*

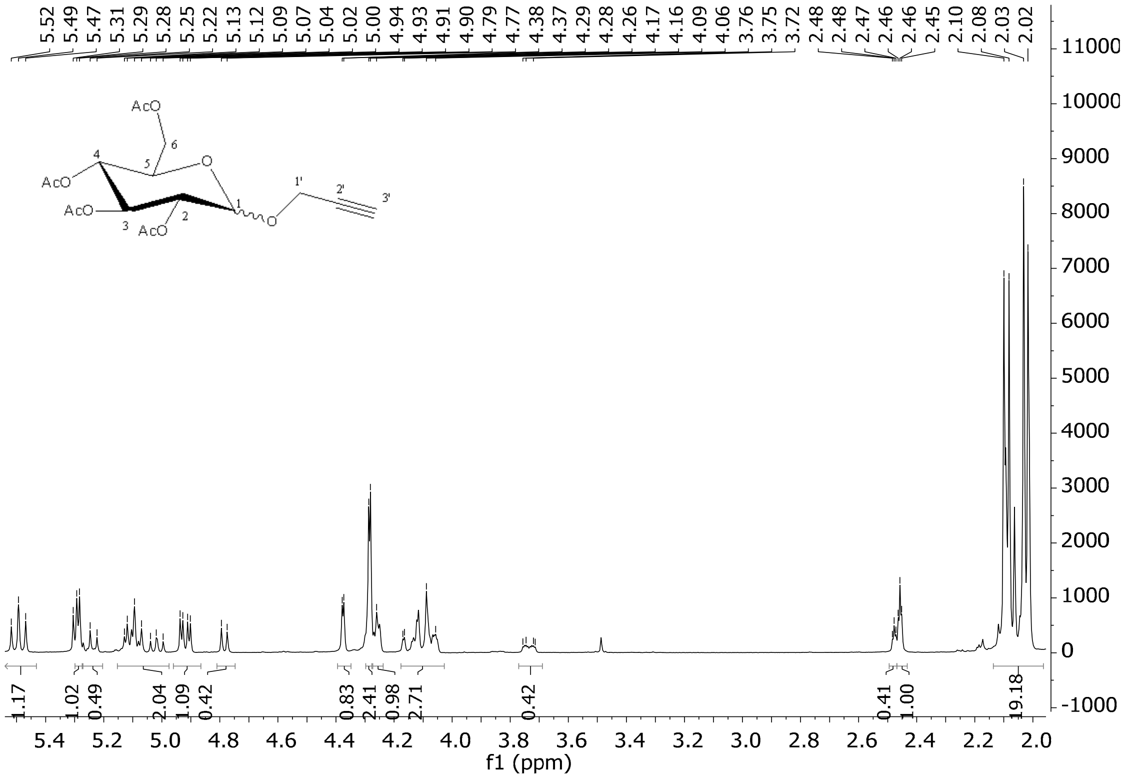

S14- ^{1}H NMR (400 MHz, $CDCl_3$-d_6) spectrum of compound **12**.

*1-[1′-Ethylamine-triazolyl-4]-1-methyl-2,3,4,6-tetra-O-acetyl-D-glucopyranoside (**13**)*

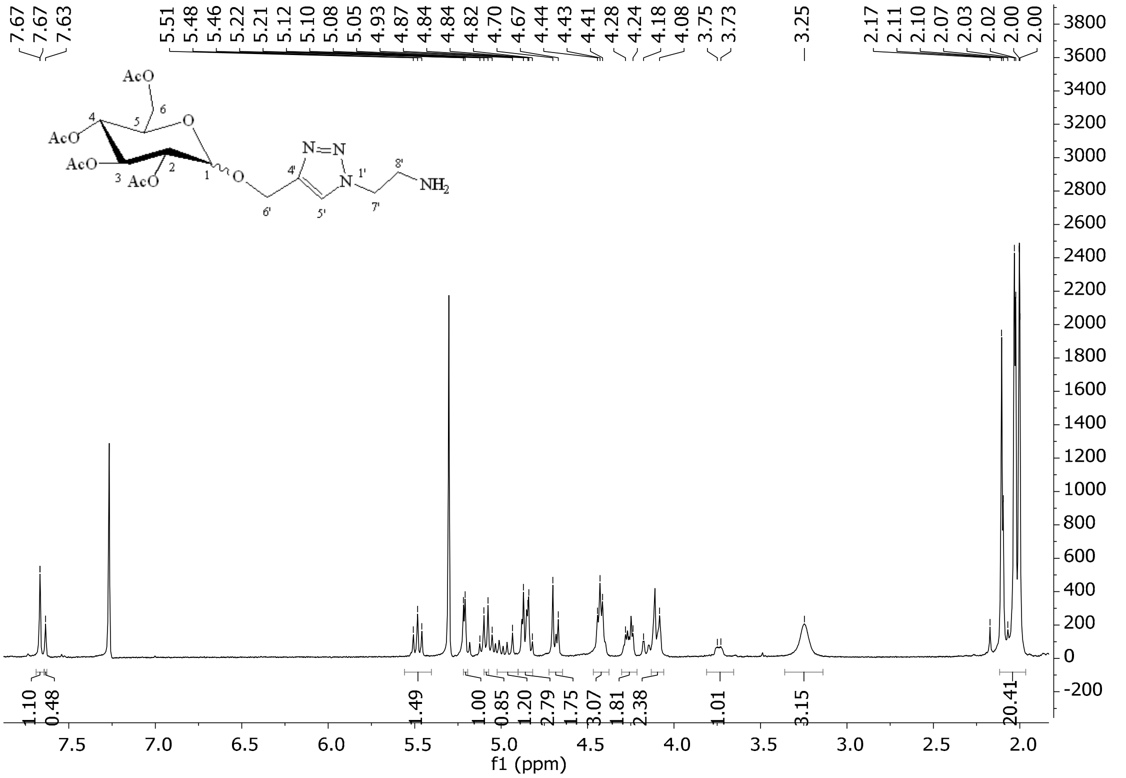

S15- ^{1}H NMR (400 MHz, $CDCl_3$) spectrum of compound **13**.

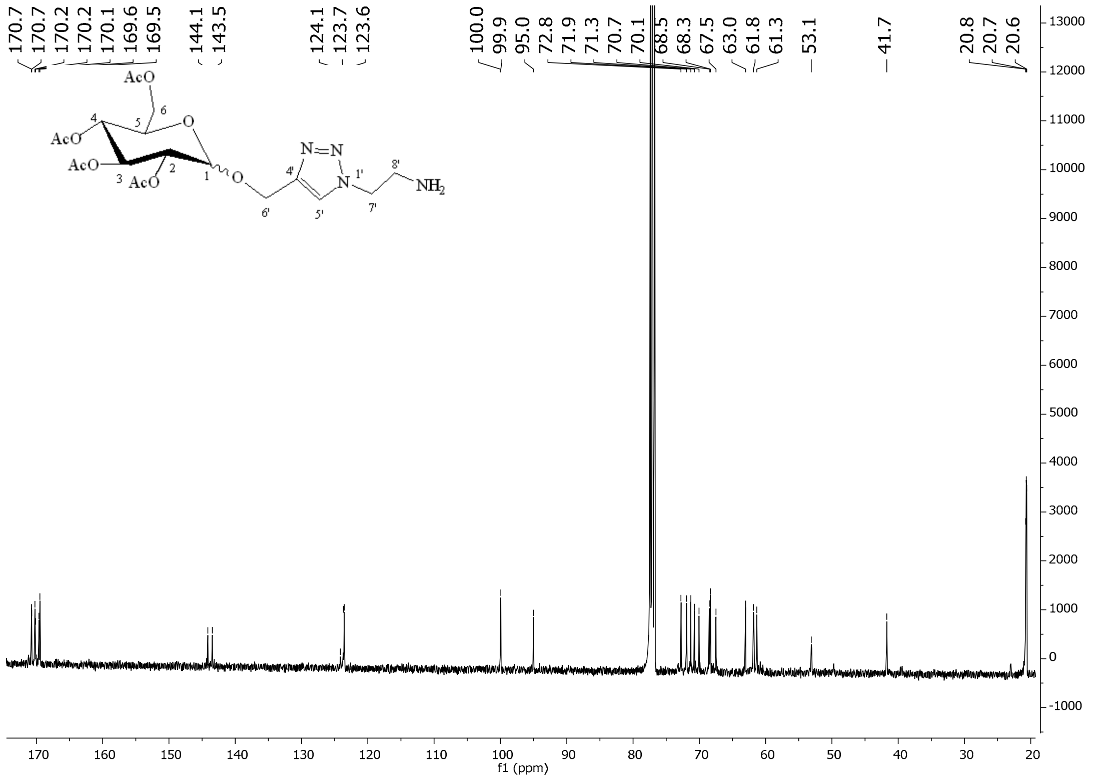

S16- ^{13}C NMR (101 MHz, $CDCl_3$) spectrum of compound **13**.

*5′-O-[(4-Methylphenyl)sulfonyl] thymidine (**15**)*

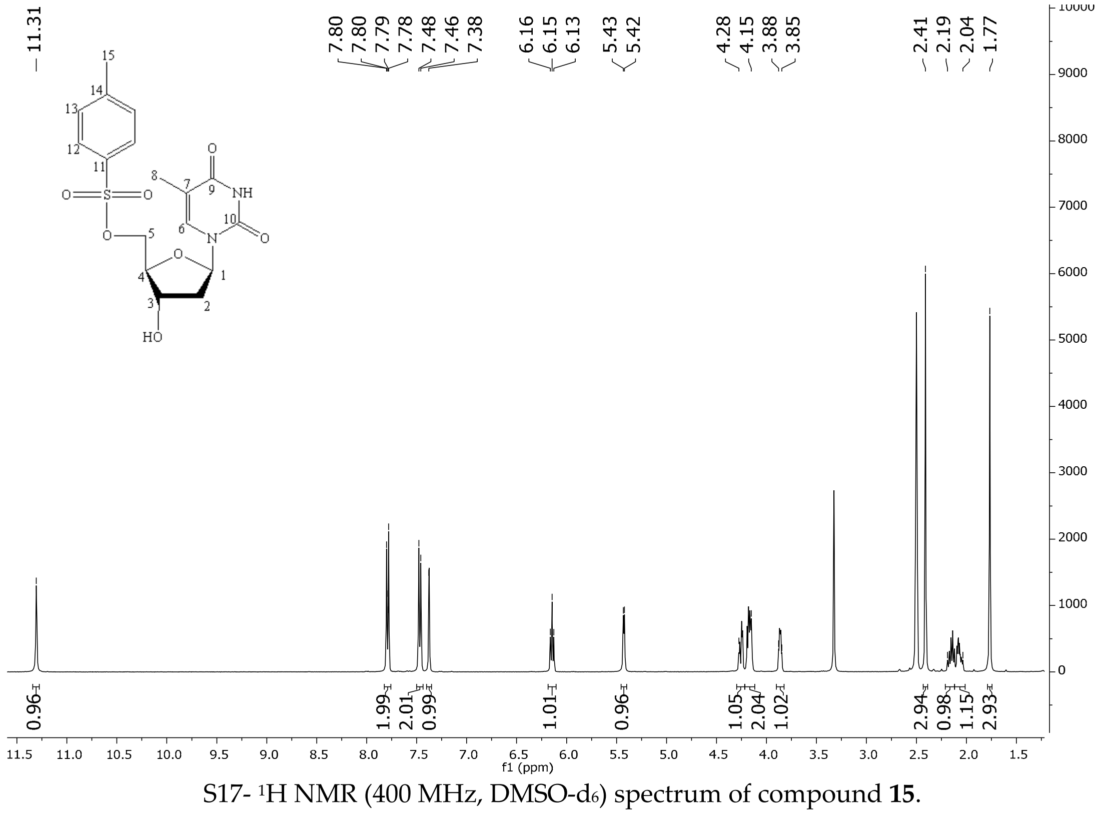

S17- ^{1}H NMR (400 MHz, DMSO-d_6) spectrum of compound **15**.

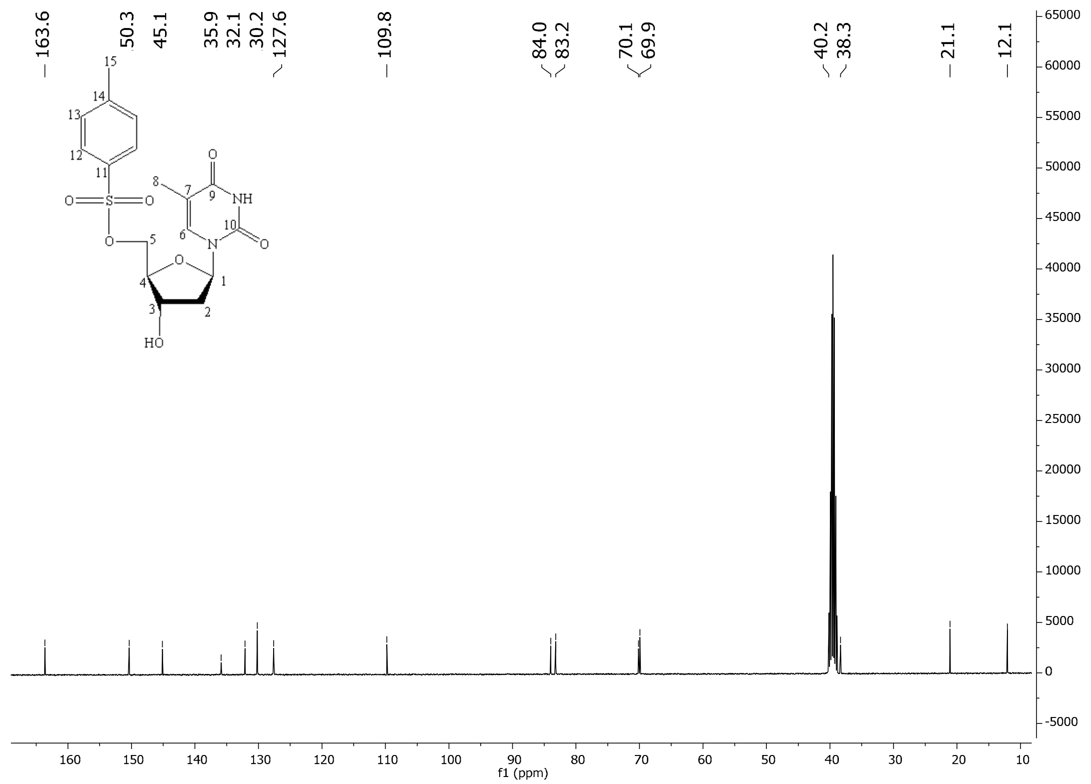

S18- ^{13}C NMR (101 MHz, DMSO-d_6) spectrum of compound **15**.

*5′-Azide-5′-deoxythymidine (**16**)*

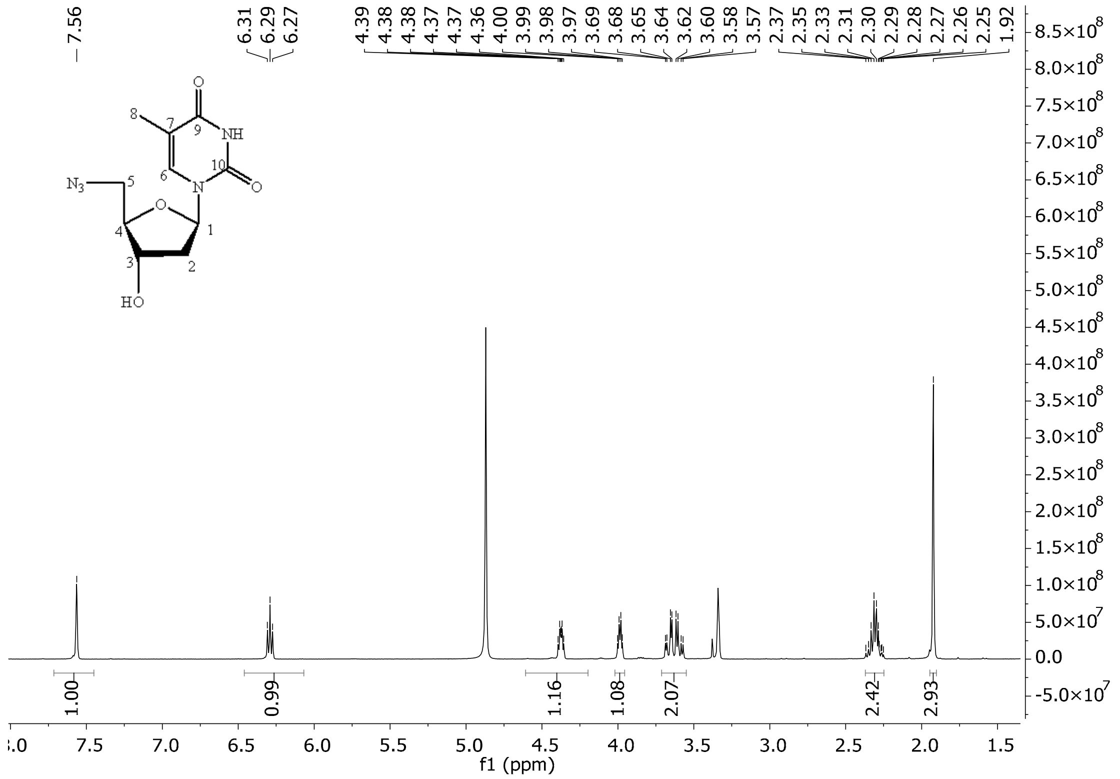

S19- ^1H NMR (400 MHz, CD_3OD) spectrum of compound **16**.

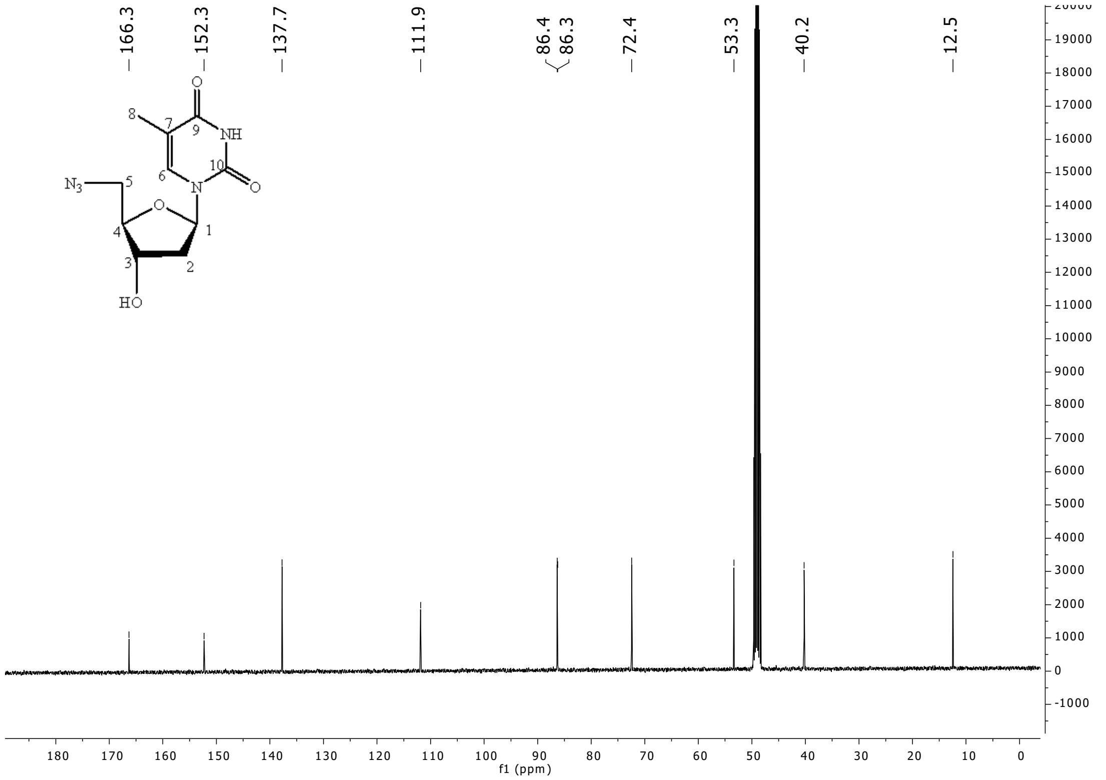

S20- ^{13}C NMR (101 MHz, CD_3OD) spectrum of compound **16**.

*α-Coumarin 3-carboxylate-ω-hydroxyl PEG$_{1000}$ (**17**)*

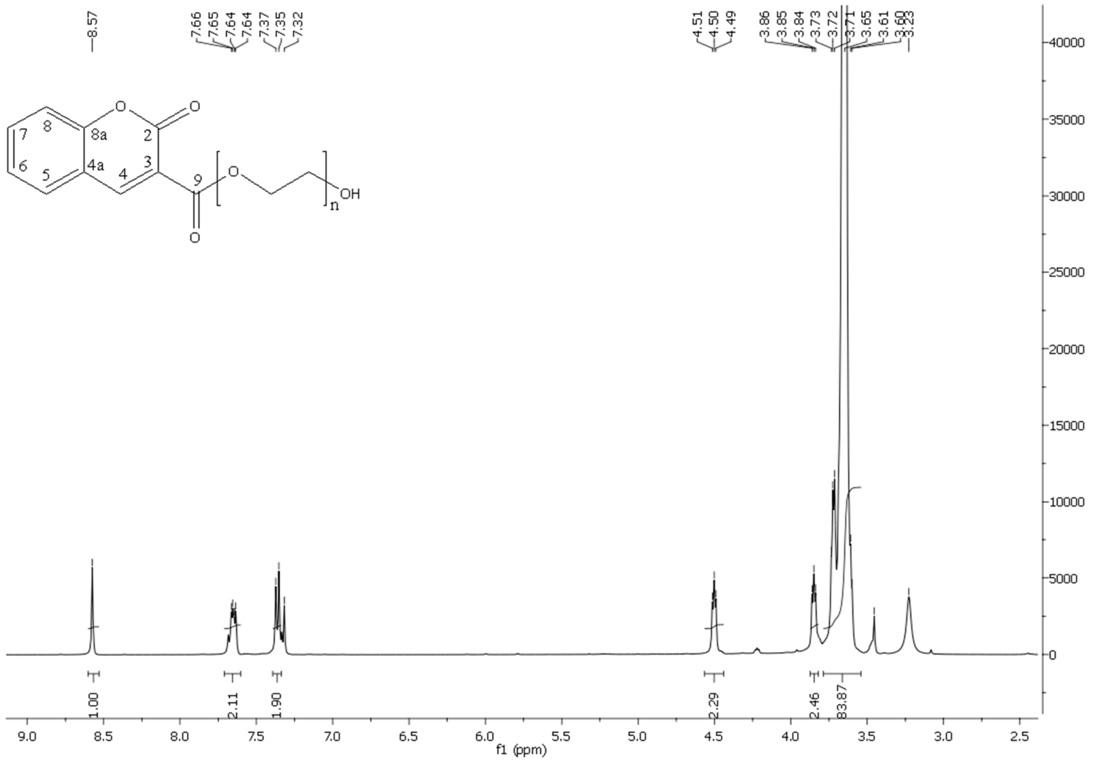

S21- ^{1}H NMR (400 MHz, $CDCl_3$) spectrum of compound **17**.

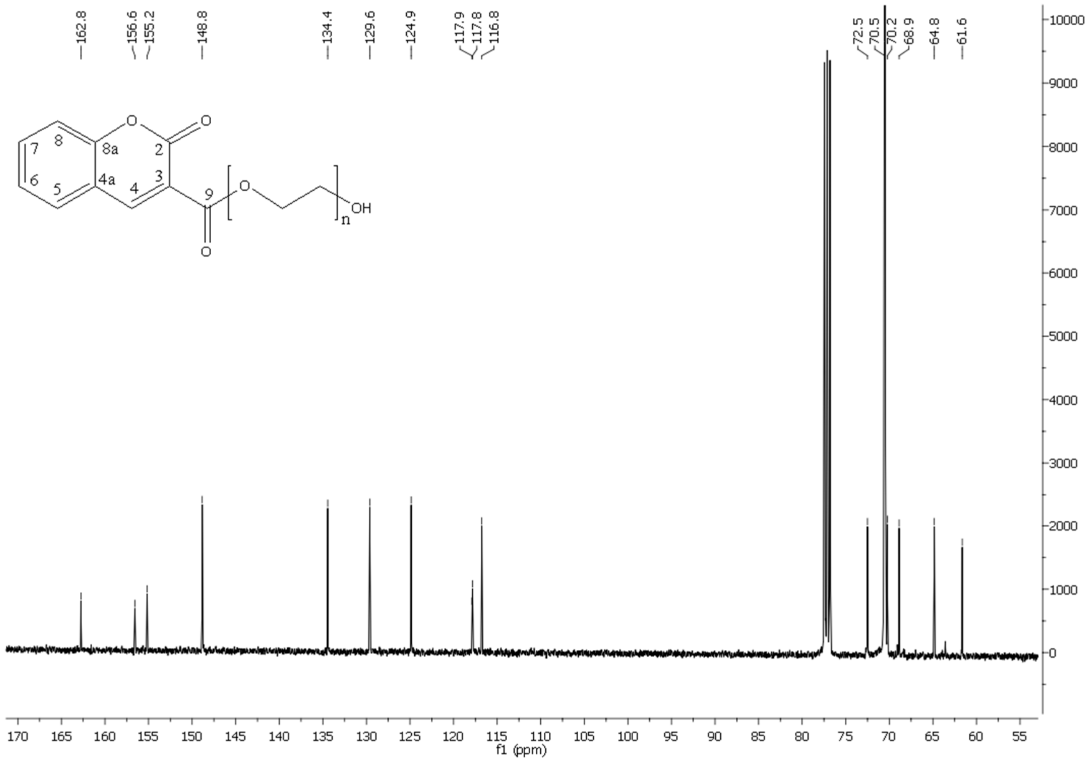

S22- ^{13}C NMR (101 MHz, $CDCl_3$) spectrum of compound **17**.

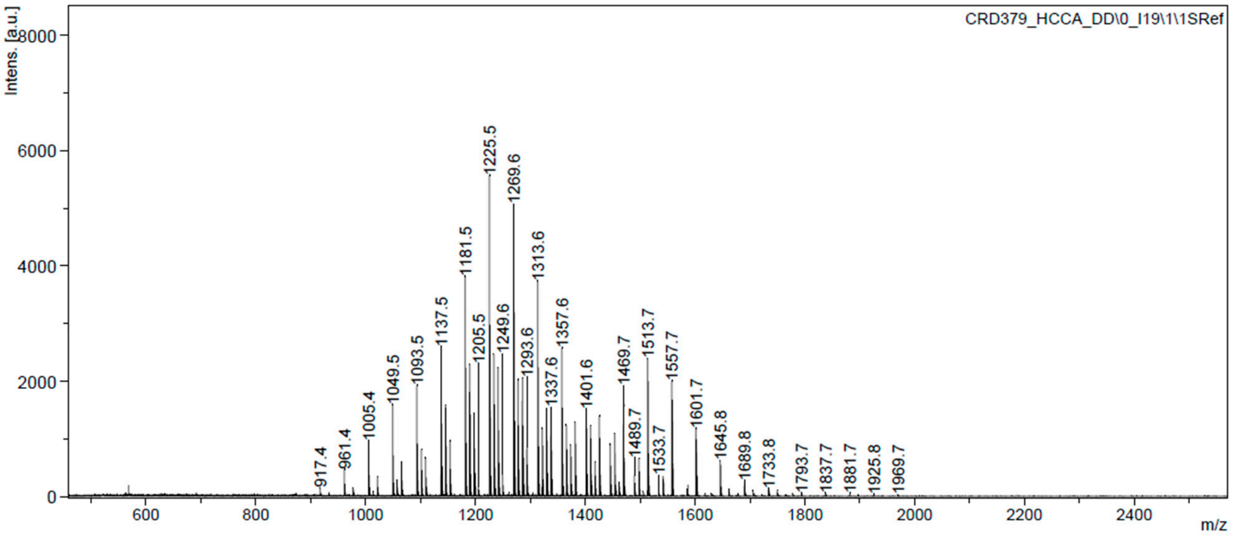

S23- MALDI-TOF spectrum of compound **17**.

*α-Coumarin 3-carboxylate-ω-tosyl PEG1000 (**18**)*

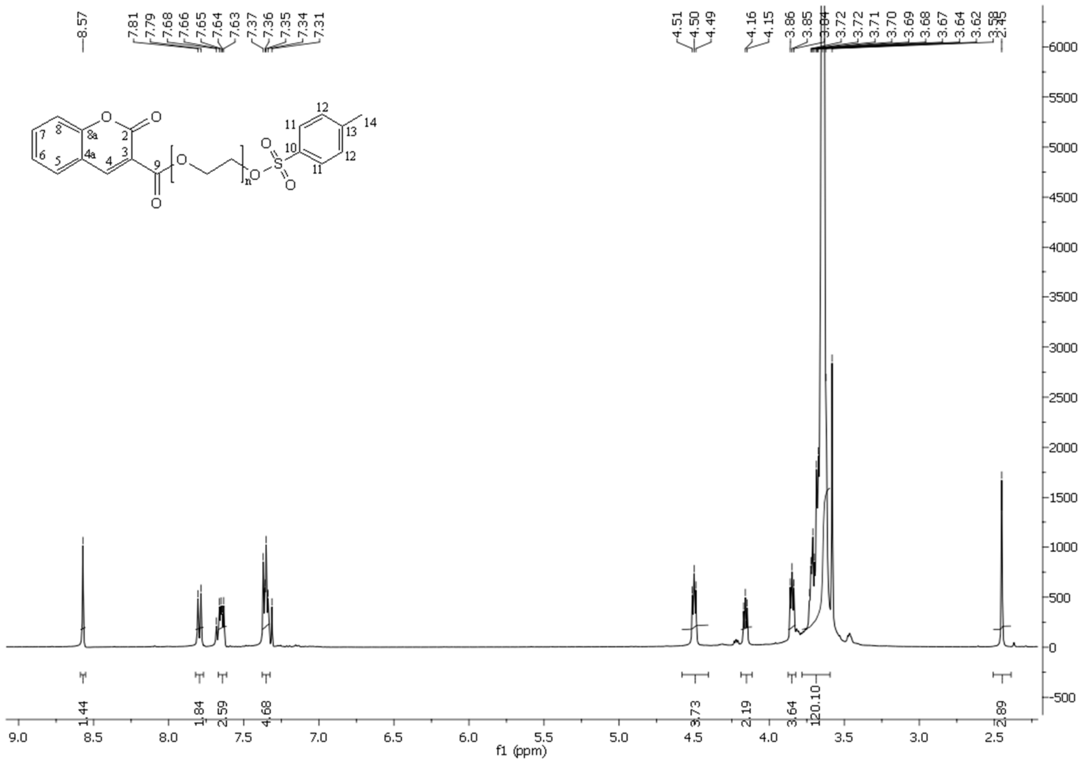

S24- ^{1}H NMR (400 MHz, $CDCl_3$) spectrum of the compound **18**.

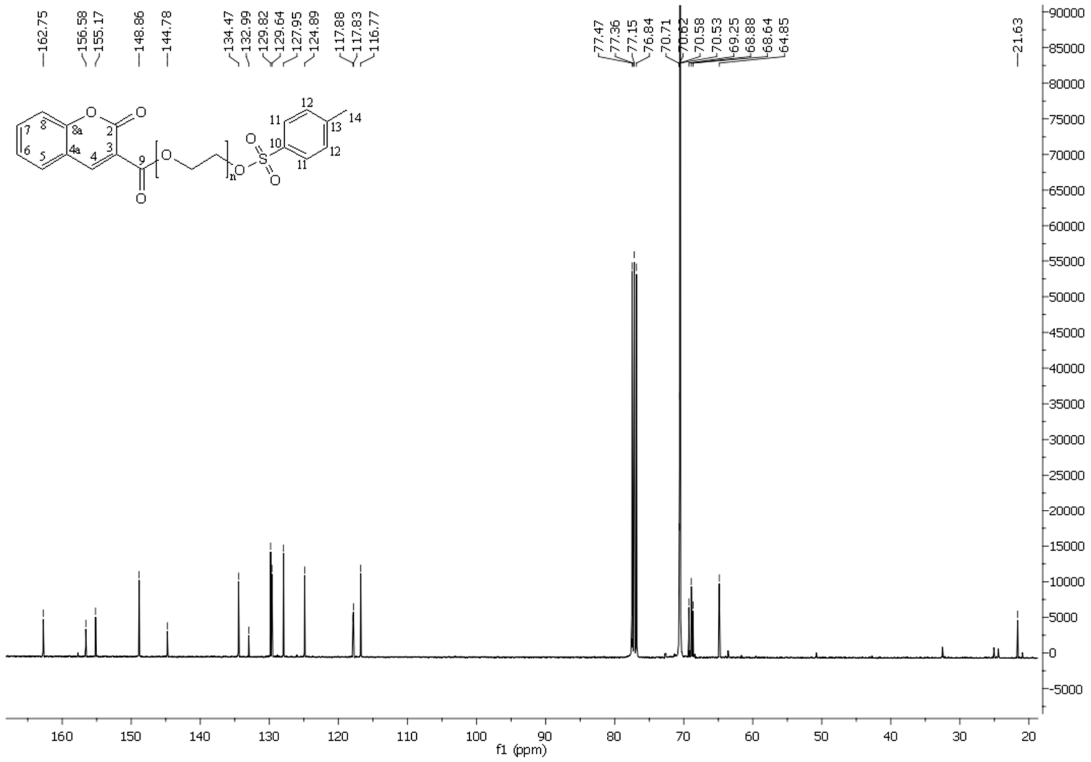

S25- ^{13}C NMR (101 MHz, $CDCl_3$) spectrum of the compound **18**.

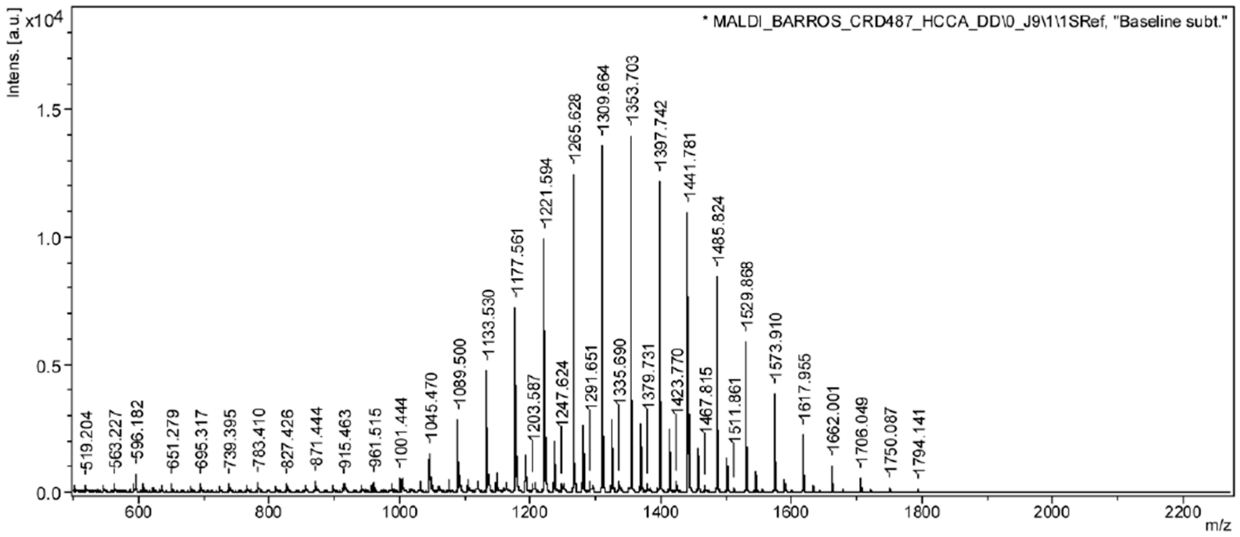

S26- MALDI-TOF spectrum of compound **18**.

*α-Coumarin 3-carboxylate-ω-azide PEG1000 (**19**)*

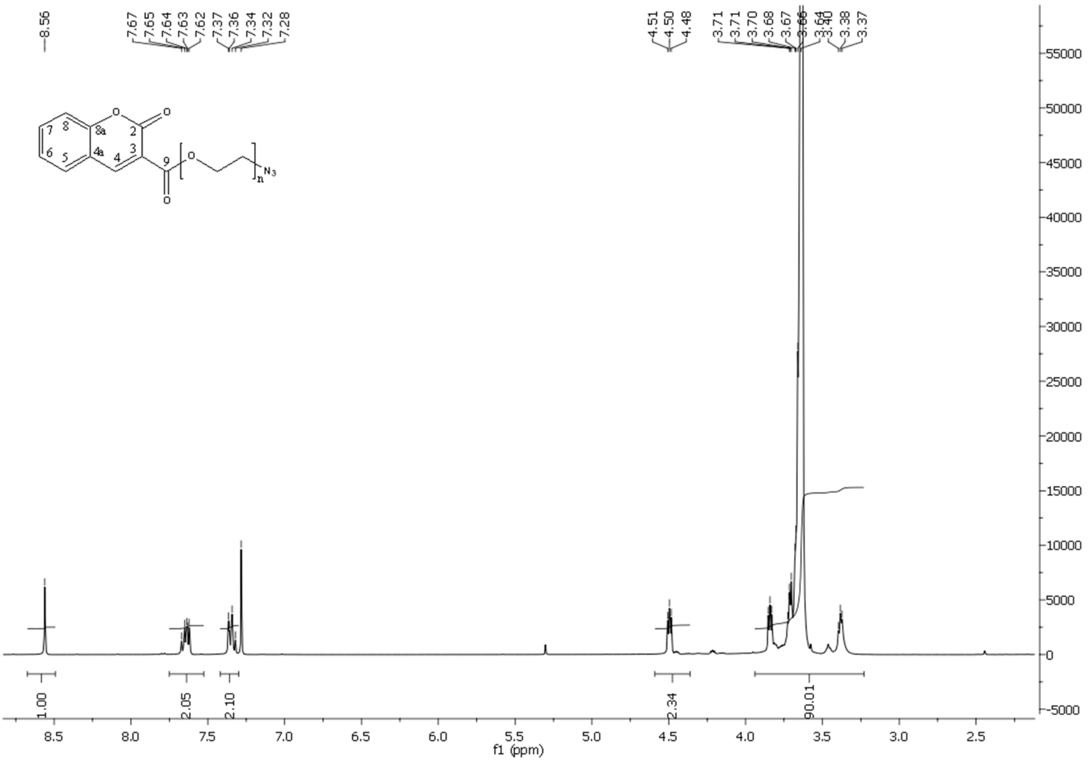

S27- ^{1}H NMR (400 MHz, $CDCl_3$) spectrum of the compound **19**.

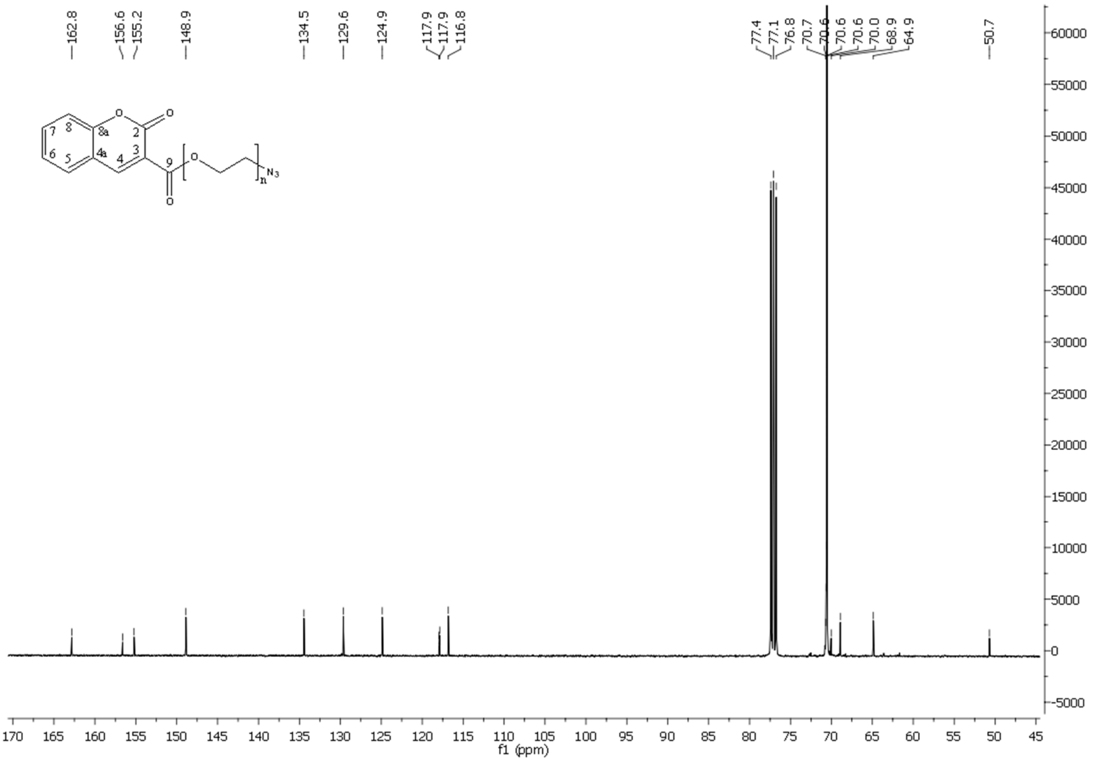

S28- ^{13}C NMR (101 MHz, $CDCl_3$) spectrum of the compound **19**.

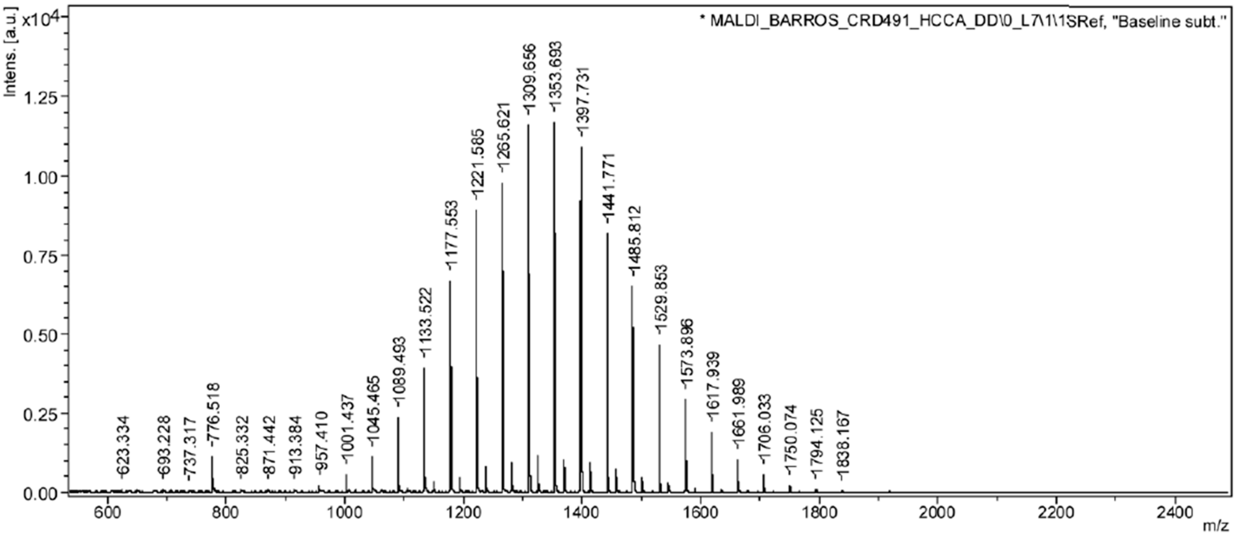

S29- MALDI-TOF spectrum of compound **19**.

*α-Coumarin 3-carboxylate-ω-[(1*H*-1,2,3-triazol-4-yl)methoxygalactopyranosyl] PEG$_{1000}$ (**20**)*

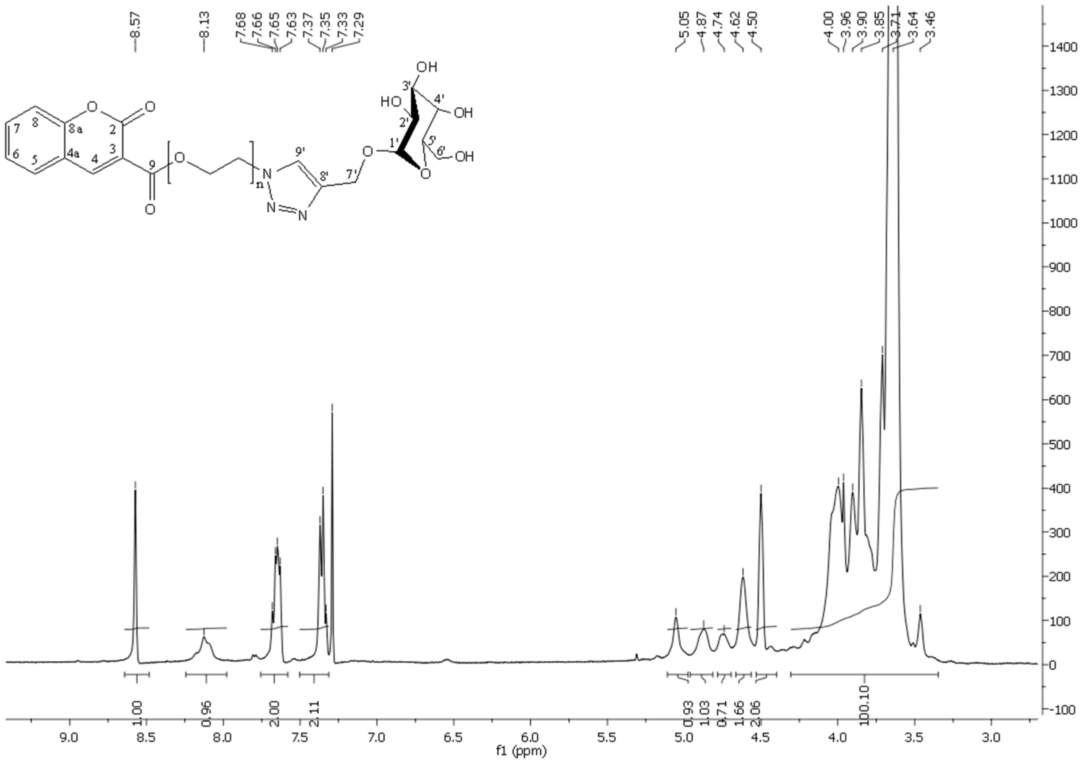

S30- ^{1}H NMR (400 MHz, $CDCl_3$) spectrum of the compound **20**.

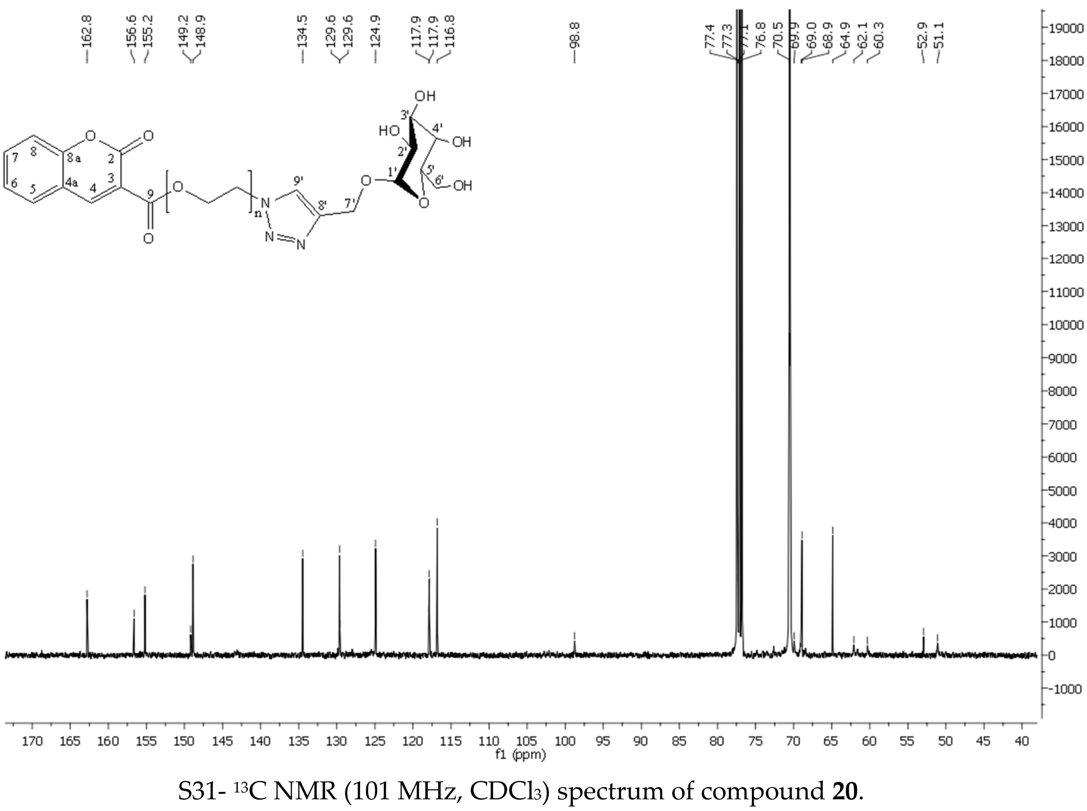

S31- ^{13}C NMR (101 MHz, $CDCl_3$) spectrum of compound **20**.

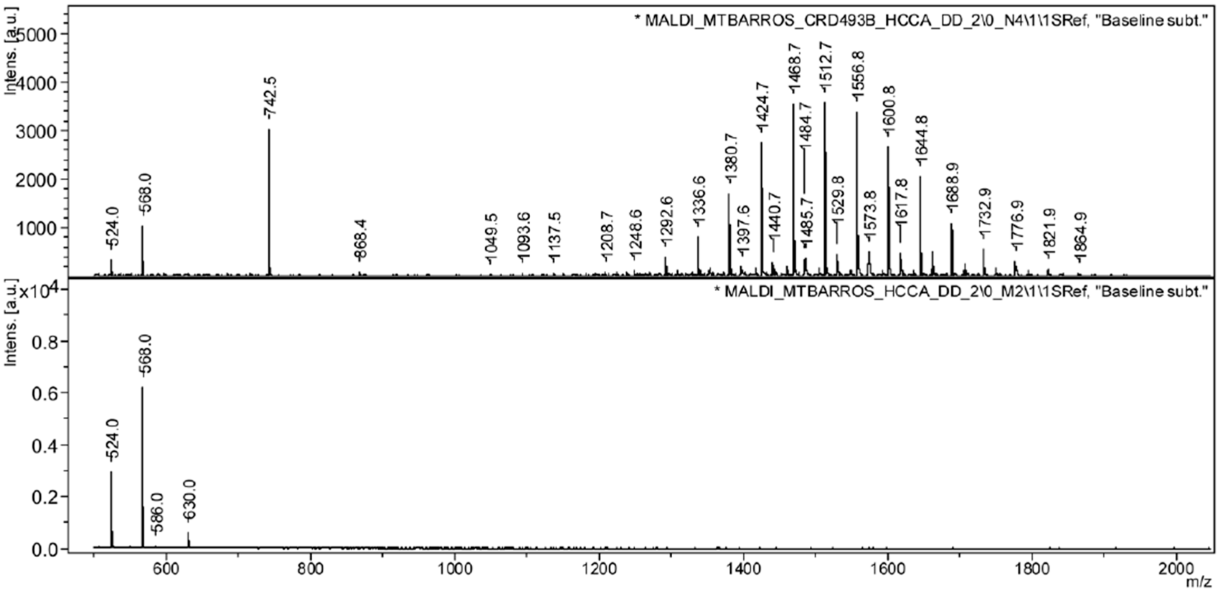

S32- MALDI-TOF spectrum of compound **20**.

*α-Tosyl-ω-tosyl PEG$_{1000}$ (**21**)*

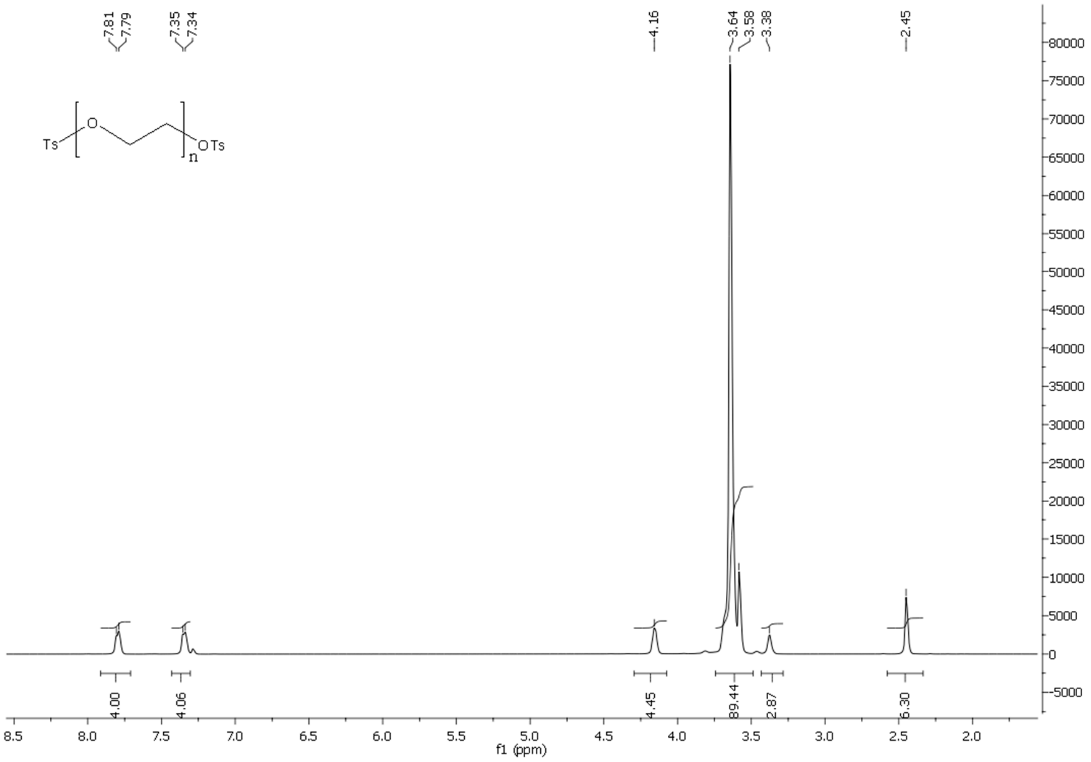

S33- ^{1}H NMR (400 MHz, CDCl$_3$) spectrum of compound **21**.

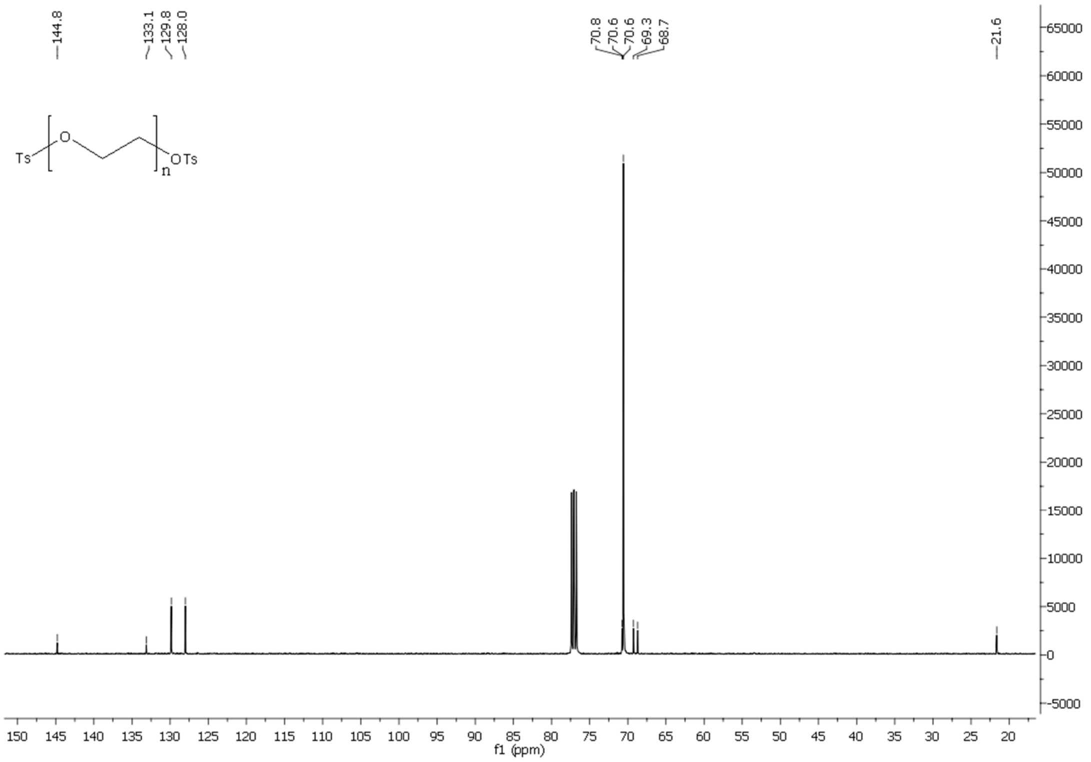

S34- ^{13}C NMR (101 MHz, $CDCl_3$) spectrum of compound **21**.

*α-Azide-ω-azide PEG$_{1000}$ (**22**)*

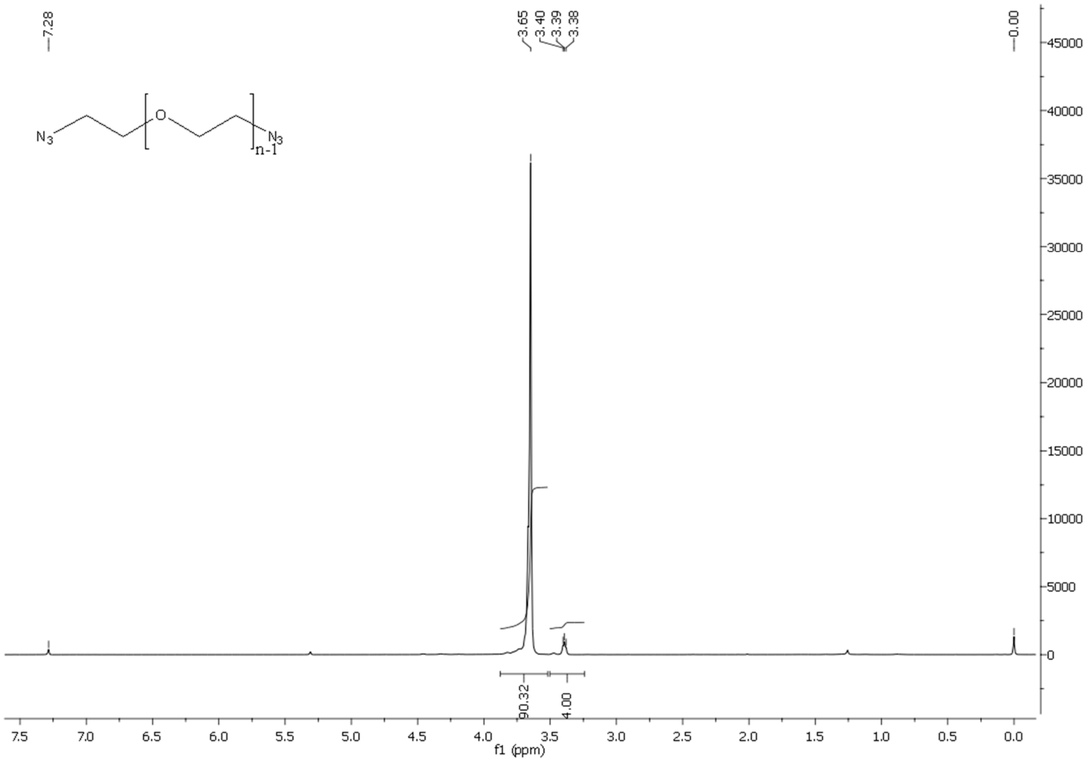

S35- ^{1}H NMR (400 MHz, $CDCl_3$) spectrum of compound **22**.

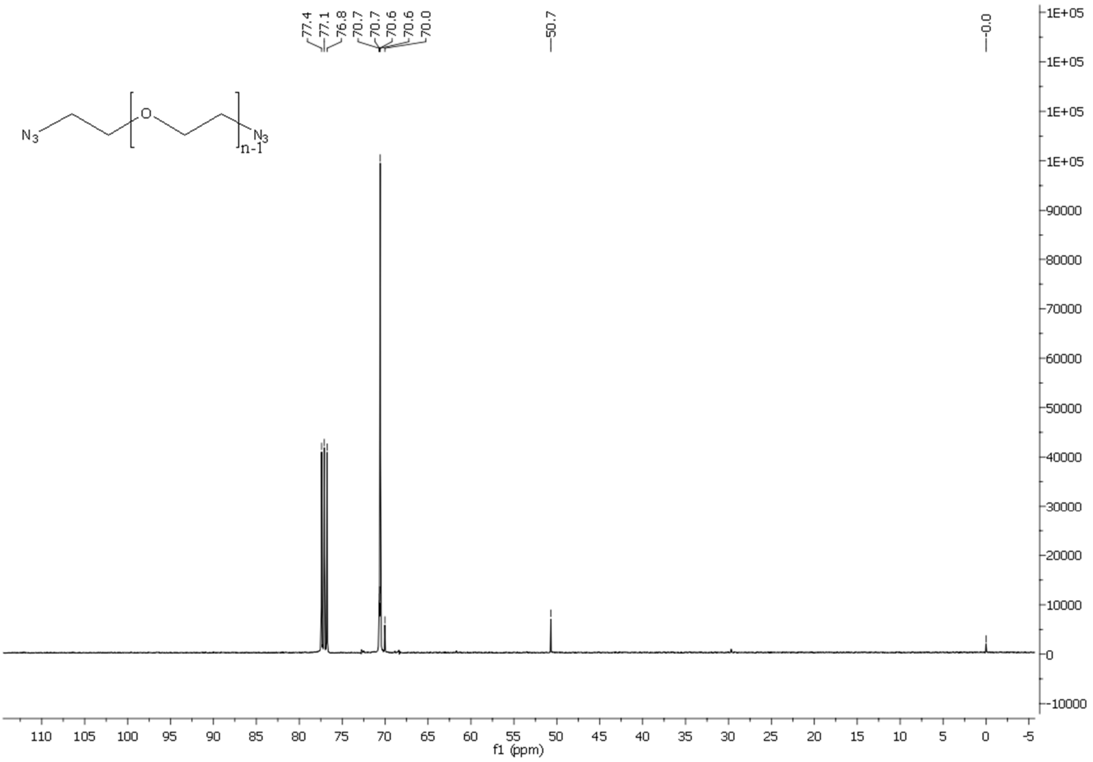

S36- ^{13}C NMR (101 MHz, $CDCl_3$) spectrum of compound **22**.

*α-Azide-ω-[7-((1*H*-1,2,3-triazol-4-yl)methoxy)-2*H*-chromen-2-one)] PEG$_{1000}$ (**23**)*

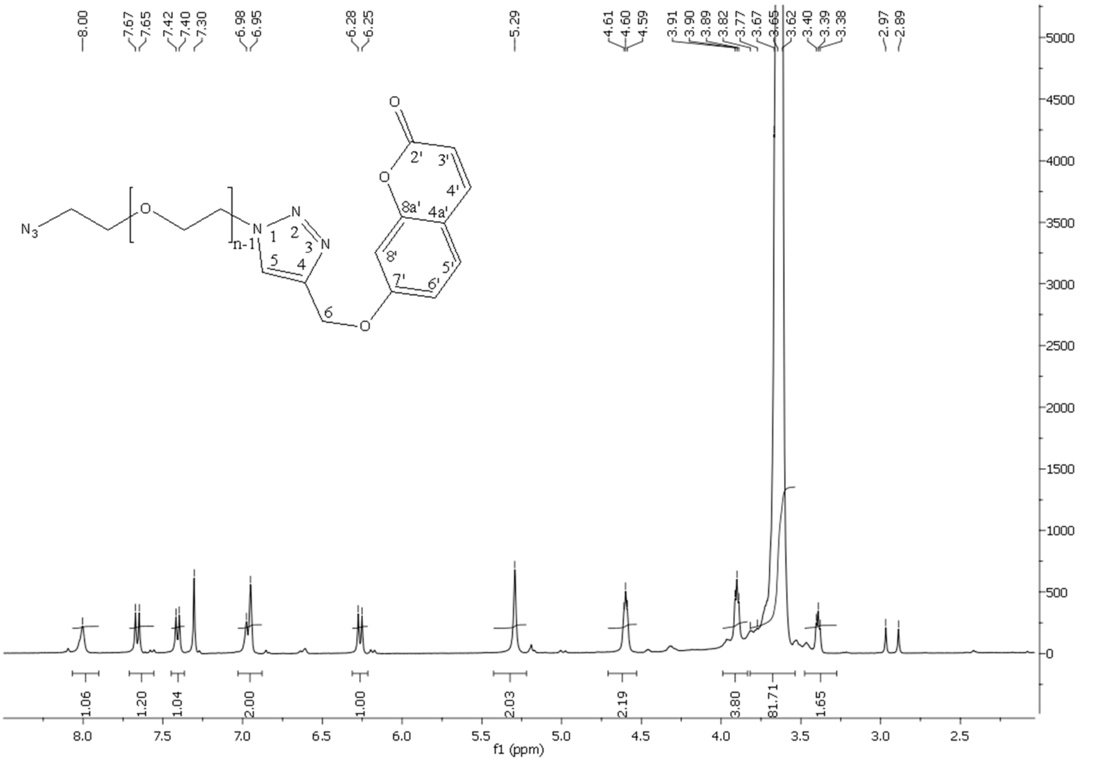

S37- ^{1}H NMR (400 MHz, $CDCl_3$) spectrum of compound **23**.

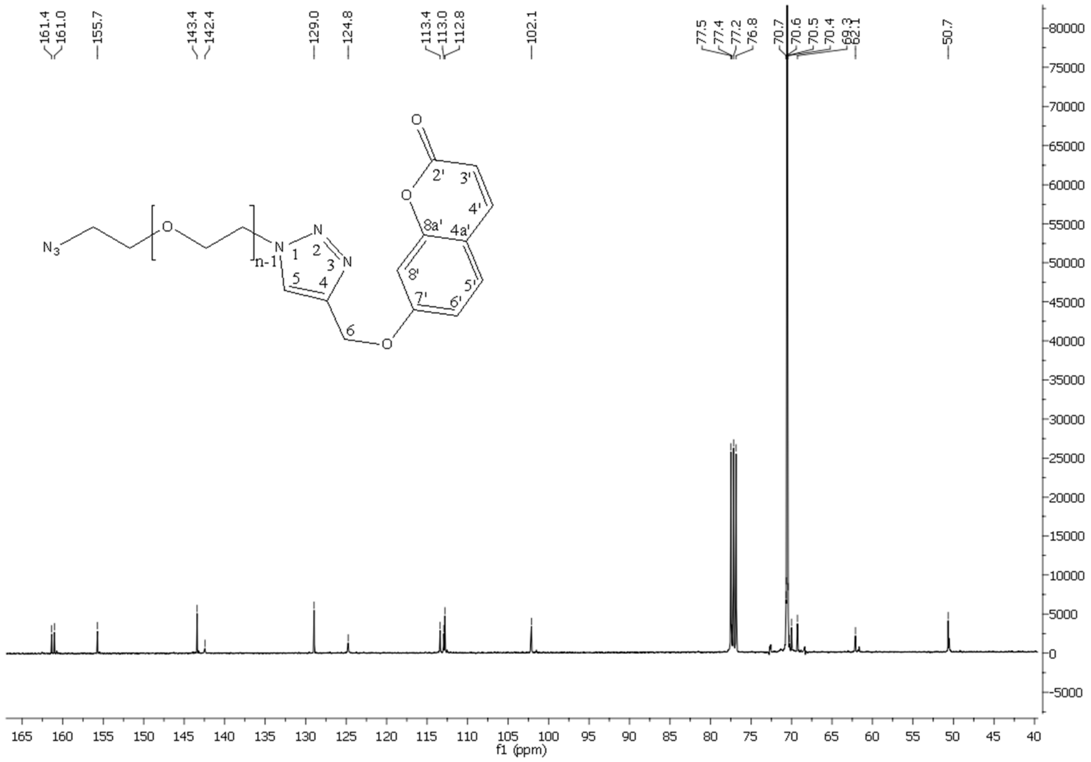

S38- ^{13}C NMR (101 MHz, $CDCl_3$) spectrum of compound **23**.

α–[7-((1*H*-1,2,3-triazol-4-yl)methoxy)-2*H*-chromen-2-one)]–ω–[(1*H*-1,2,3-triazol-4-yl)methoxygalactopyranosyl] PEG_{1000} (**24**)

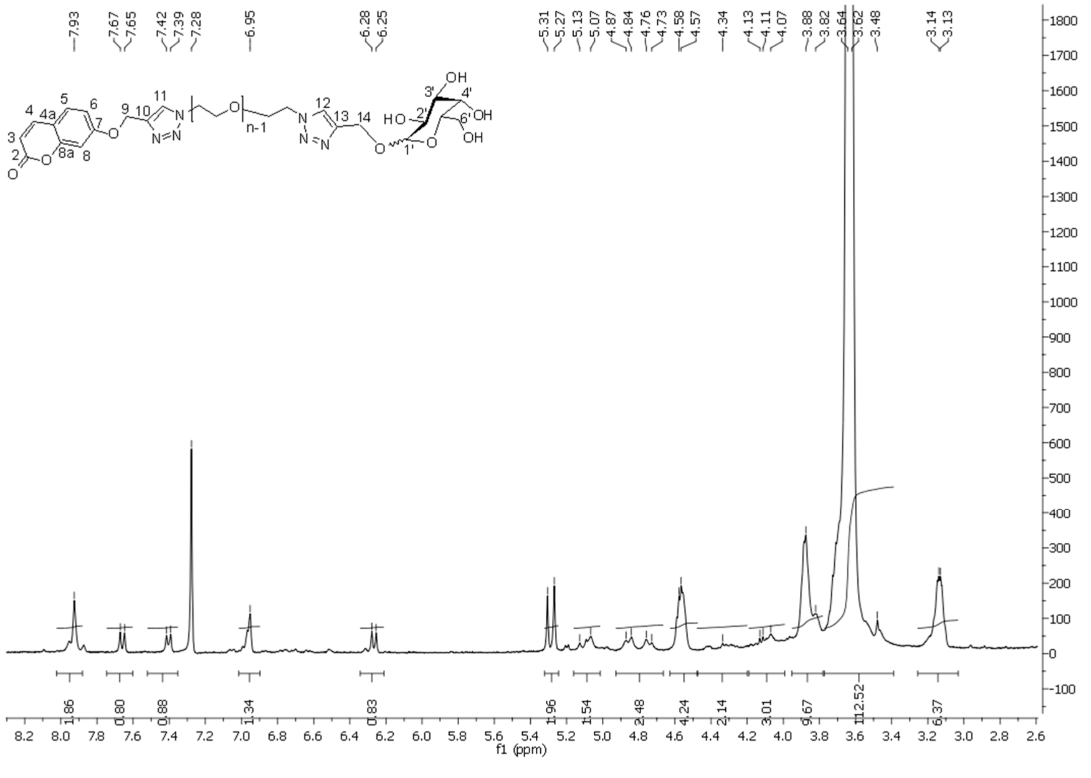

S39- ^{1}H NMR (400 MHz, $CDCl_3$) spectrum of compound **24**.

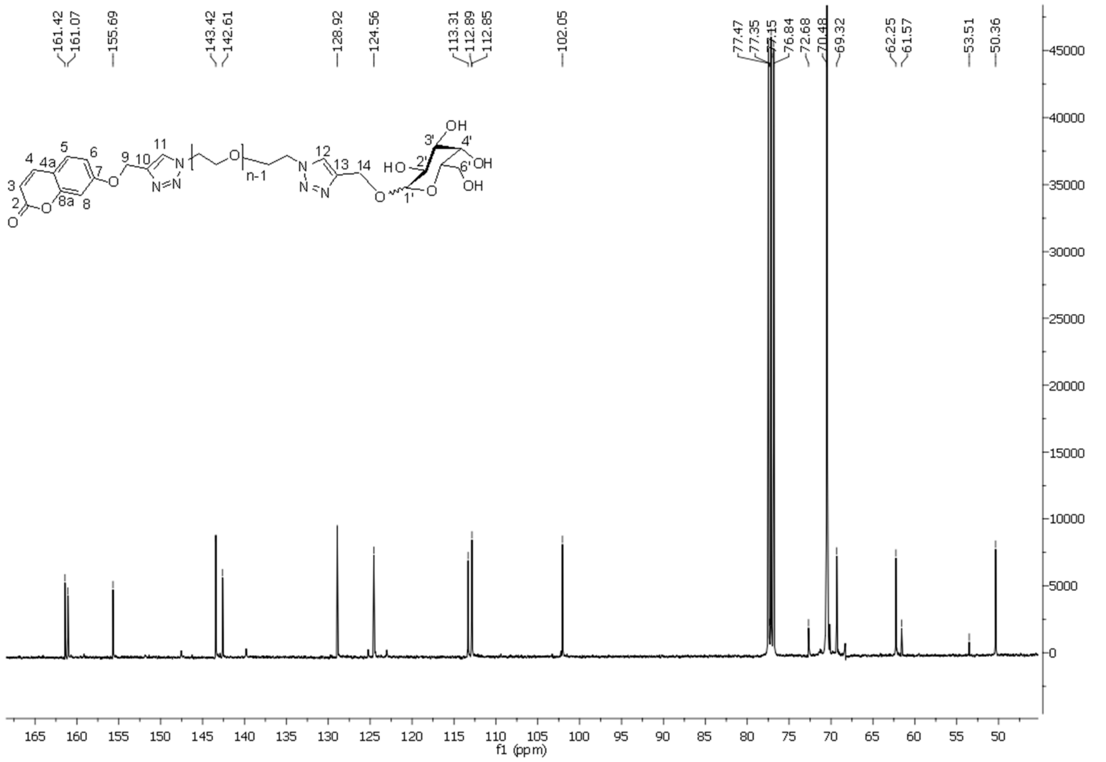

S40- ^{13}C NMR (101 MHz, $CDCl_3$) spectrum of compound **24**.

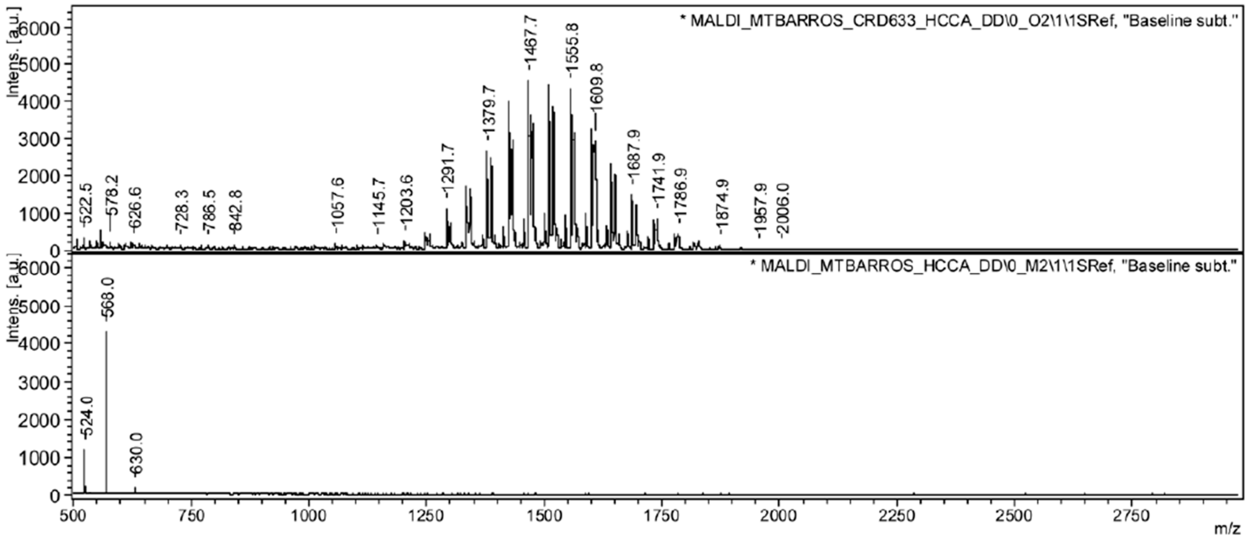

S41- MALDI-TOF spectrum of compound **24**.

α–[7-((1*H*-1,2,3-triazol-4-yl)methoxy)-2*H*-chromen-2-one)]–*ω*–[(1*H*-1,2,3-triazol-4-yl)methoxymannopyranosyl] PEG1000 (**25**)

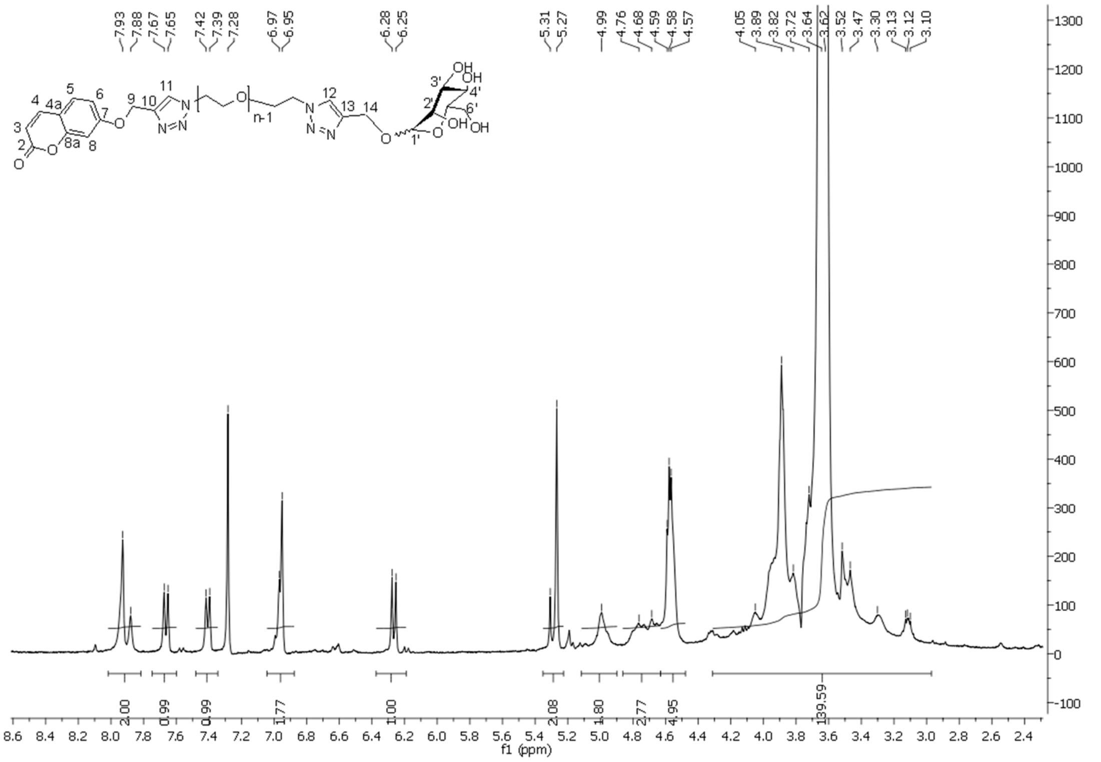

S42- ^{1}H NMR (400 MHz, $CDCl_3$) spectrum of compound **25**.

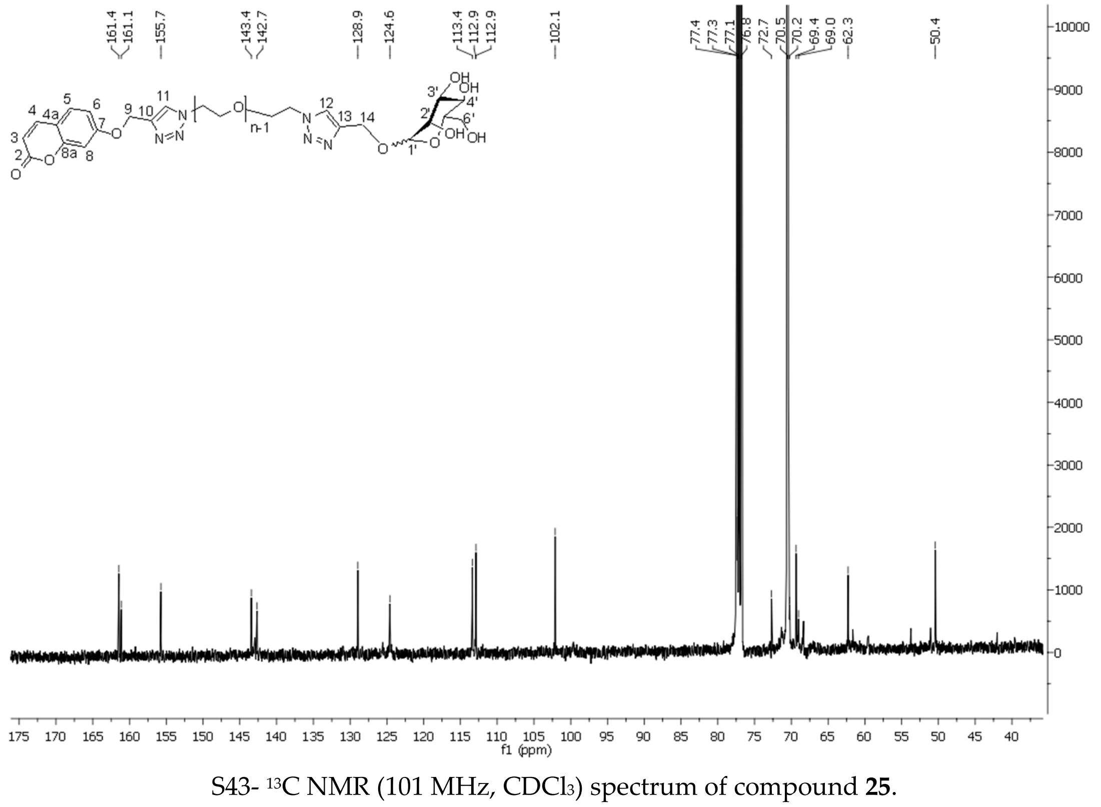

S43- ^{13}C NMR (101 MHz, $CDCl_3$) spectrum of compound **25**.

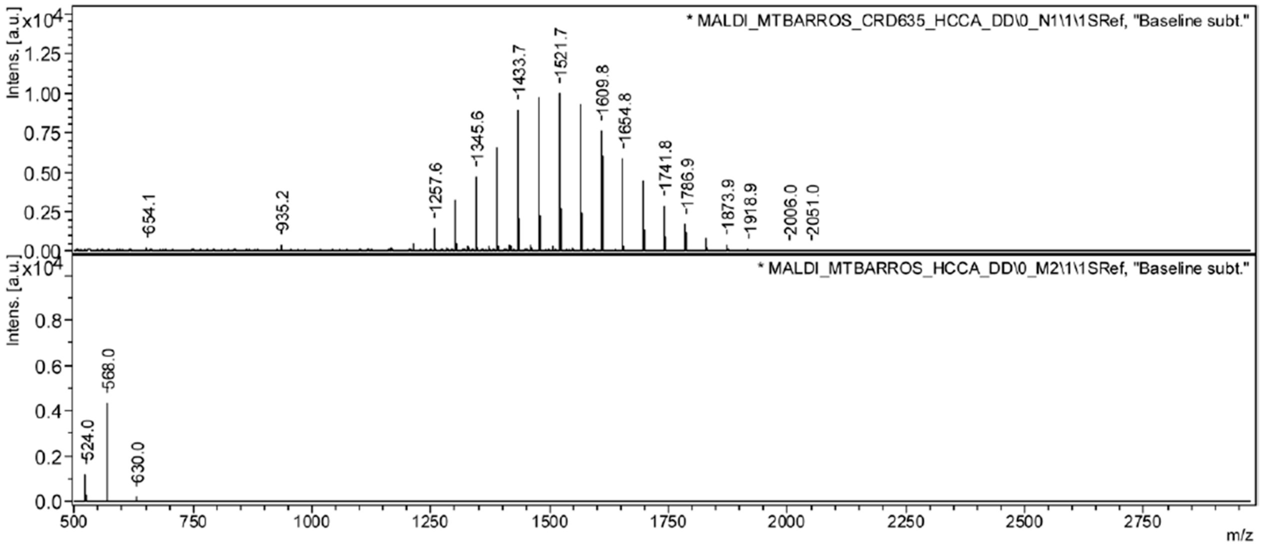

S44- MALDI-TOF spectrum of compound **25**.

*α-3-[3-(4,8-dimethyl]-7-(2-propynyloxy)]-coumarinyl propanoate-ω-3-[3-(4,8-dimethyl]-7-(2-propynyloxy)]-coumarinyl propanoate PEG1000 (**26**)*

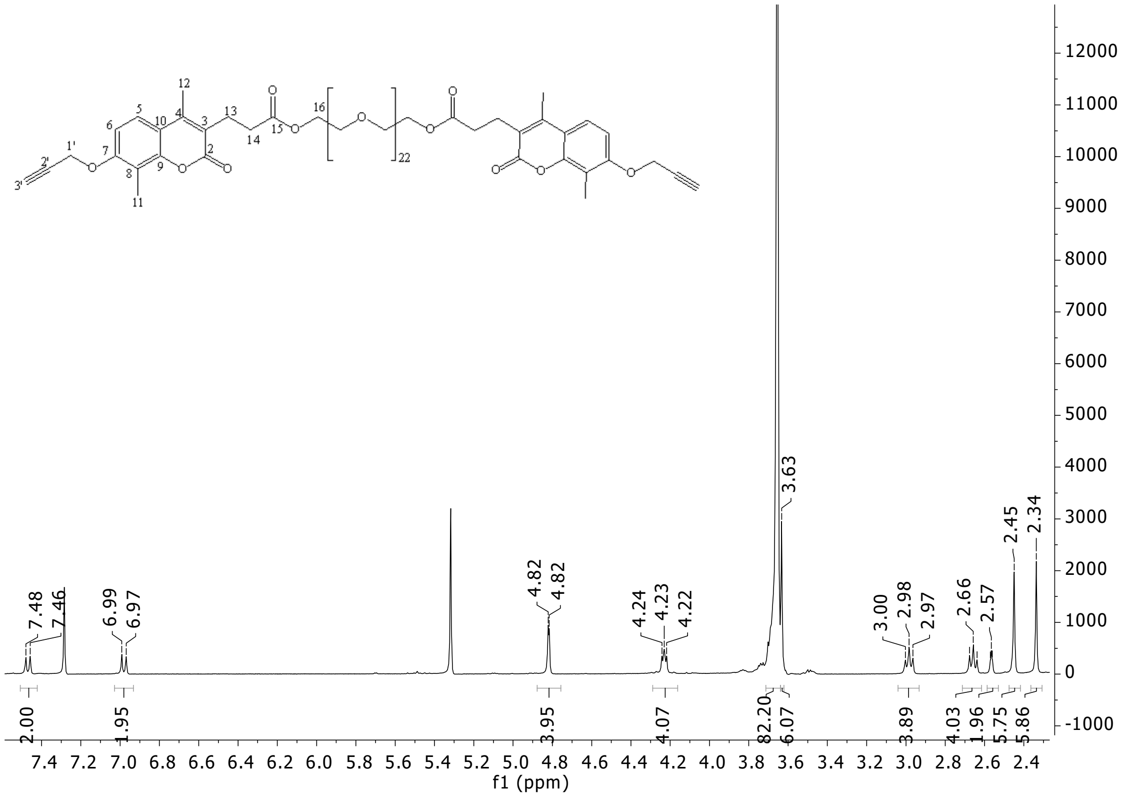

S45- ^{1}H NMR (400 MHz, $CDCl_3$) spectrum of compound **26**.

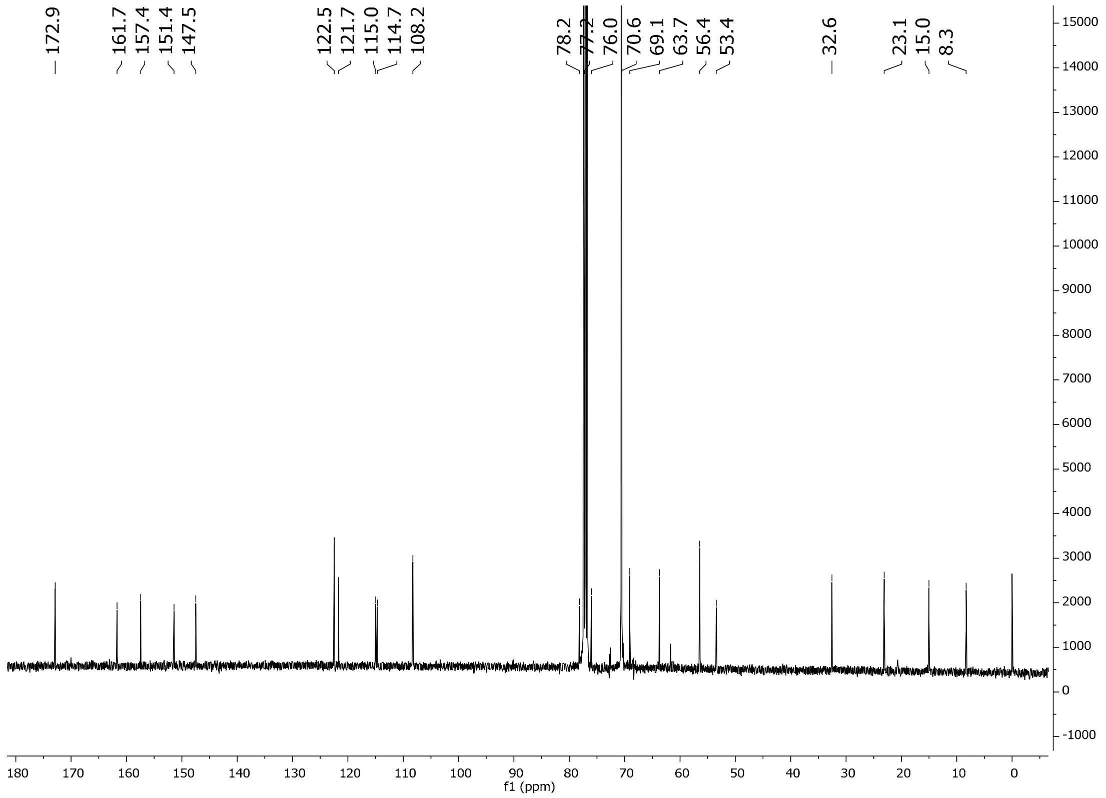

S46- ^{13}C NMR (101 MHz, $CDCl_3$) spectrum of compound **26**.

*α- thymidinyl-5-((1,2,3-triazol-1-yl)-3-[3-(4,8-dimethyl)-7-(methyloxi)-cumarinyl)propanoate -ω- thymidinyl-5-((1,2,3-triazol-1-yl)-3-[3-(4,8-dimethyl)-7-(methyloxi)-cumarinyl)propanoate PEG$_{1000}$ (**27**)*

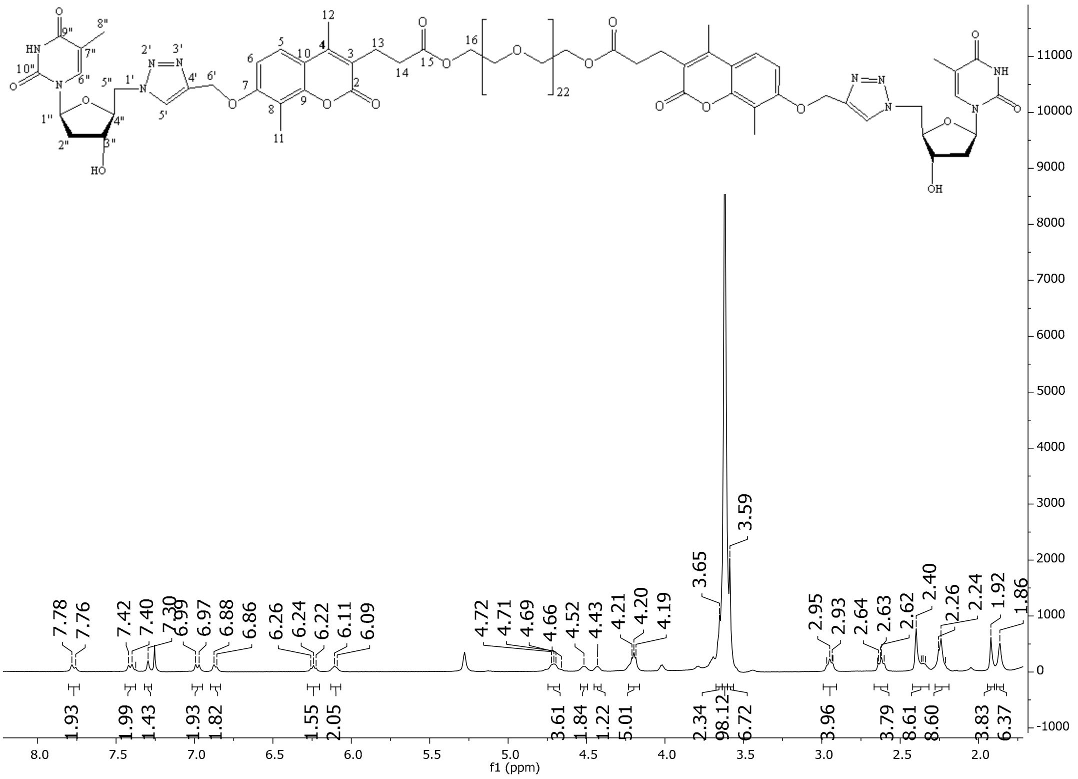

S47- ^{1}H NMR (400 MHz, $CDCl_3$) spectrum of compound **27**.

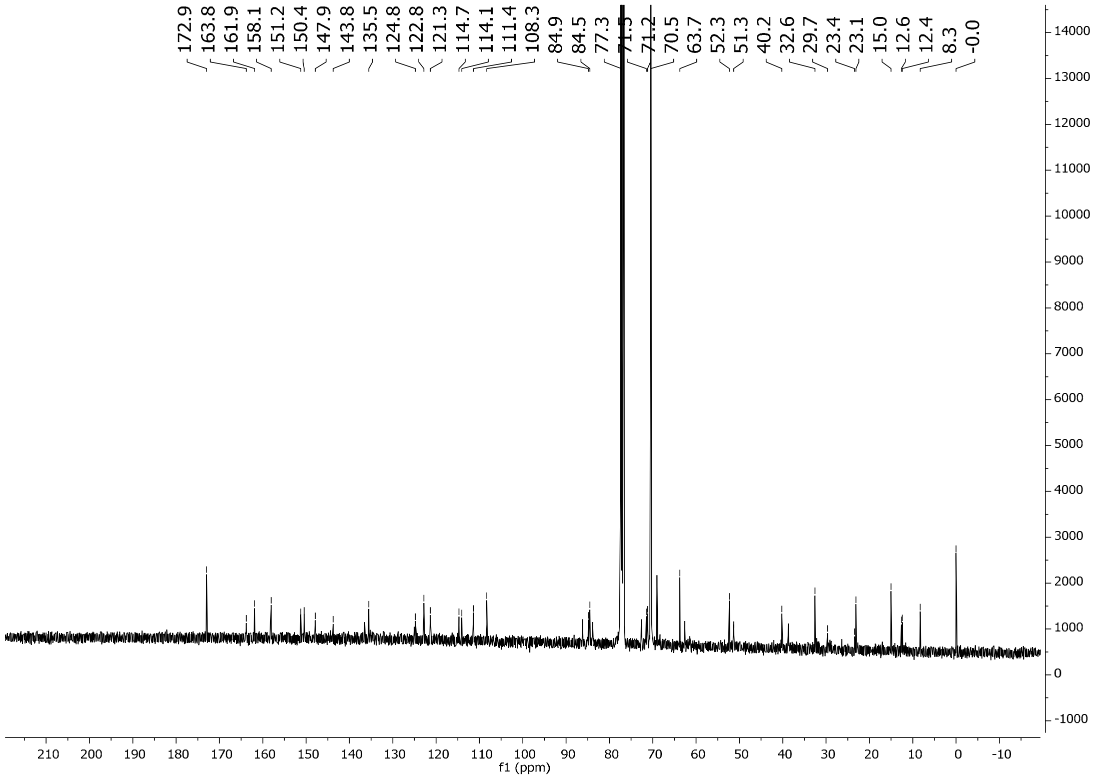

S48- ^{13}C NMR (101 MHz, $CDCl_3$) spectrum of compound **27**.

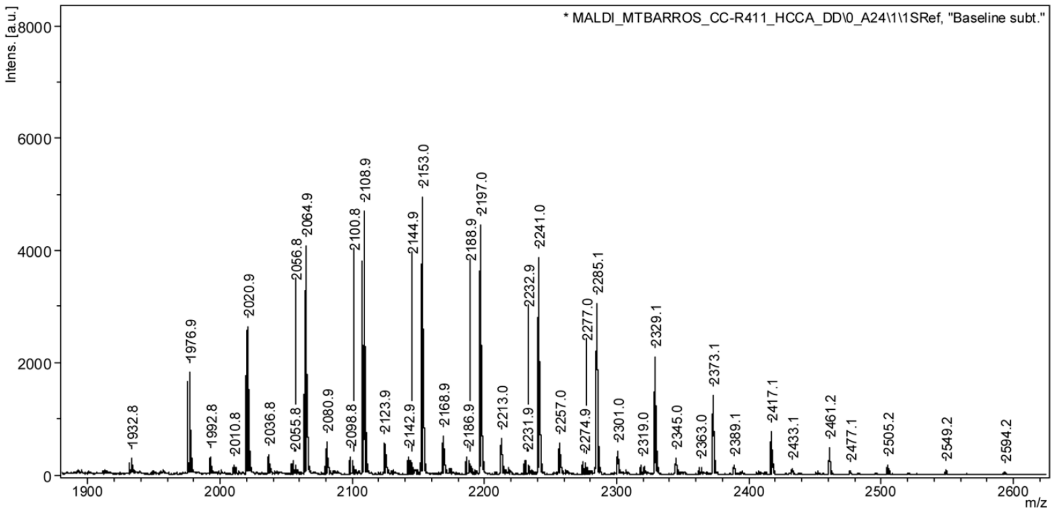

S49- MALDI-TOF spectrum of compound **27**.

*1-[1′-Ethylamide-triazolyl-4]-1-O-methyl-D-glucopyranoside-PLGA conjugate (**28**)*

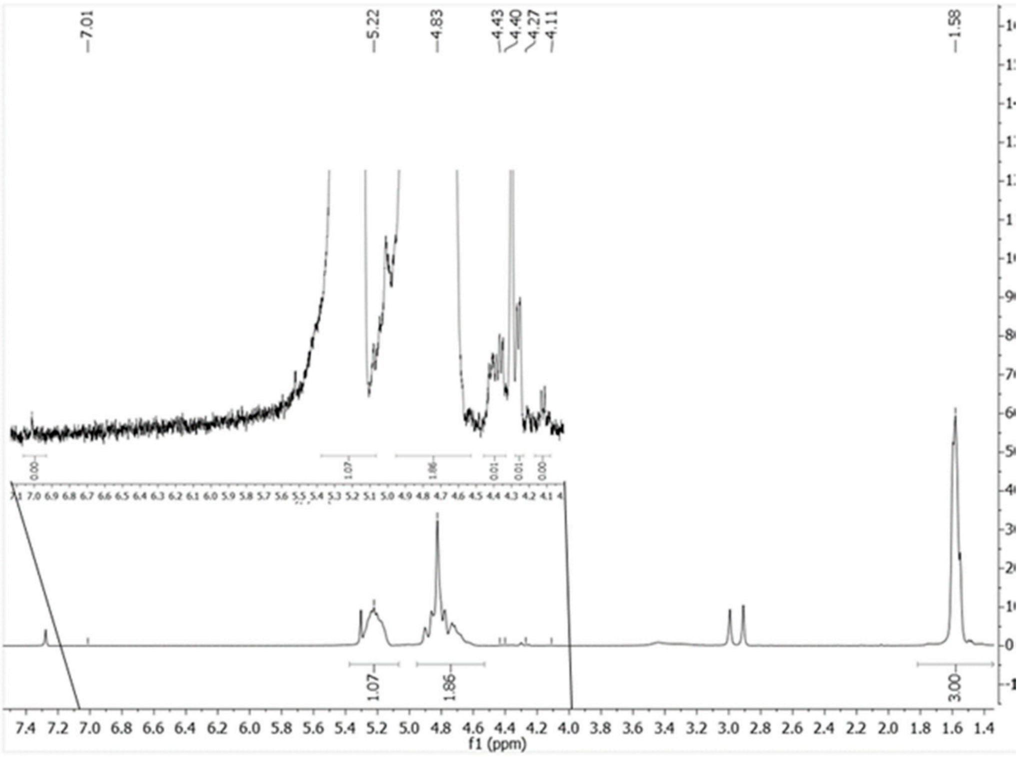

S50- ^{1}H NMR (400 MHz, $CDCl_3$) spectrum of compound **28**.

*7-((1-(2-Amide-ethyl)-1*H*-1,2,3-triazol-4-yl)methoxy)-4-methyl-2*H*-chromen-2-one -PLGA conjugate (**29**)*

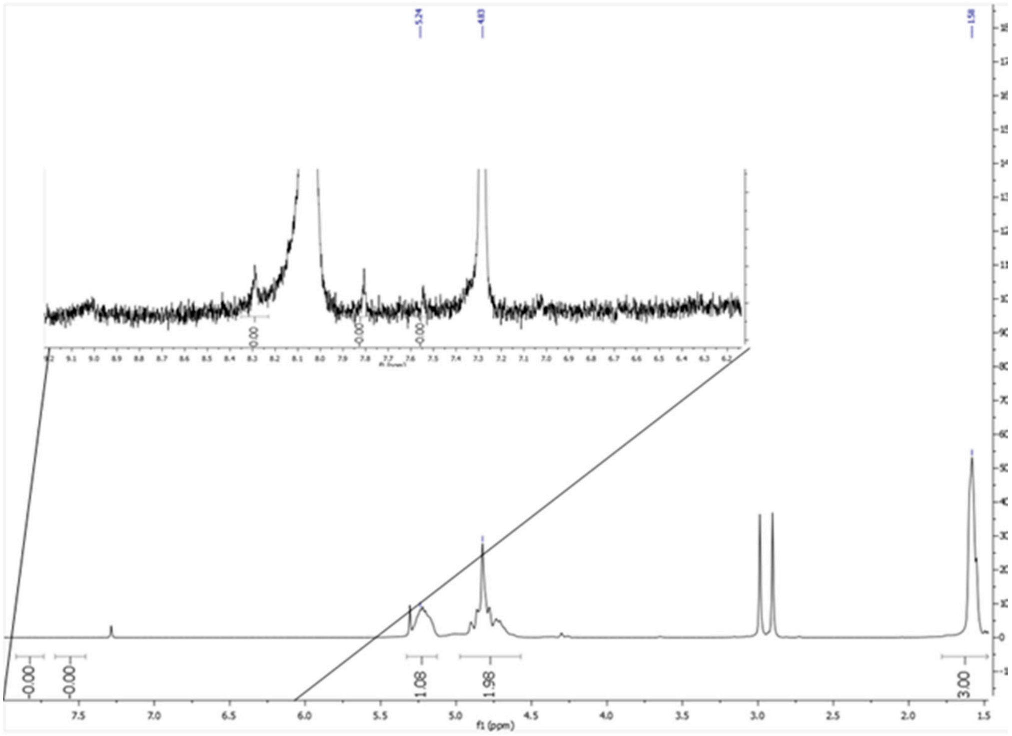

S51- ^{1}H NMR (400 MHz, $CDCl_3$) spectrum of compound **29**.
